# KLF5 enhances CXCL12 transcription in adipose-derived stem cells to promote endothelial progenitor cells neovascularization and accelerate diabetic wound healing

**DOI:** 10.1186/s11658-025-00702-0

**Published:** 2025-03-04

**Authors:** Yunjia Xie, Xuejun Ni, Xiaofen Wan, Nating Xu, Lu Chen, Chensheng Lin, Xi Zheng, Beichen Cai, Qian Lin, Ruonan Ke, Tao Huang, Xuefeng Hu, Biao Wang, Xiuying Shan

**Affiliations:** 1 Department of Plastic Surgery, The First Affiliated Hospital of Fujian Medical University, Fuzhou, 350005 China; 2 Department of Plastic Surgery, National Regional Medical Center, Binhai Campus of the First Affiliated Hospital, Fujian Medical University, Fuzhou, 350212 China; 3 Department of Burn and Plastic Surgery, Fujian Provincial Hospital, Fuzhou, 350001 China; 4 Fujian Key Laboratory of Developmental and Neural Biology & Southern Center for Biomedical Research, College of Life Sciences, Fujian Normal University, Fuzhou, 350117 Fujian China

**Keywords:** Adipose-derived stem cells, Endothelial progenitor cells, Diabetic wound, Neovascularization, Wound healing

## Abstract

**Background:**

Adipose-derived stem cells (ADSCs) have been shown to accelerate diabetic wound healing by promoting neovascularization, though the underlying mechanisms are not fully understood. This study aims to explore whether ADSCs influence endothelial progenitor cells (EPCs) function to enhance diabetic wound healing.

**Methods:**

Human adipose-derived stem cells (hADSCs) were isolated from patient adipose tissue and cultured under normal and high glucose (HG) conditions. RNA sequencing analyzed gene expression, while immunofluorescence validated findings in patient wound tissues. Mouse adipose-derived stem cells (ADSCs) from C57BL/6 mice were evaluated in vitro for their effects on EPCs under HG using EdU, Transwell, and tube formation assays. A diabetic mouse wound model was used to assess ADSCs therapeutic effects via digital imaging, histology, and immunofluorescence. Kruppel-like factor 5 (KLF5), identified via the JASPAR database, was confirmed by immunohistochemistry and immunofluorescence. KLF5 and C-X-C motif chemokine 12 (CXCL12) expression levels were measured by enzyme-linked immunosorbent assay (ELISA), western blot, and quantitative reverse transcription polymerase chain reaction (RT-qPCR), and their relationship was validated through dual-luciferase assays.

**Results:**

We constructed a neovascularization-related signature (NRS) comprising 75 genes on the basis of differentially expressed genes (DEGs) linked to neovascularization. GO and KEGG analyses revealed that the NRS is primarily involved in vasculature development and receptor–ligand activity. Seven hub genes (*CD34*, *CXCL12*, *FGF7*, *FGF18*,* FGF1*, *TEK*, *KIT*) were identified and validated. In a diabetic mouse model, CXCL12 knockdown in ADSCs reduced their ability of promoting wound healing and neovascularization. KLF5 expression was lower in patients with diabetic ulcers and diabetic mice wound tissues compared with normal tissues, while ADSCs treatment significantly increased KLF5 expression in diabetic mice wounds. Dual-luciferase reporter assays confirmed KLF5 as an upstream transcription factor of CXCL12. Additionally, knocking down KLF5 in ADSCs impaired their therapeutic effects on diabetic wound healing. In vitro, the addition of exogenous CXCL12 recombinant protein restored EPCs proliferation, migration, and vasculogenic capacity in a high glucose environment after KLF5 silencing in ADSCs.

**Conclusions:**

Our findings underscore the pivotal role of KLF5 in enhancing CXCL12 transcription within ADSCs, thereby facilitating EPC-mediated neovascularization and improving diabetic wound healing. Additionally, KLF5 emerges as a promising therapeutic target for accelerating tissue repair in diabetic wounds.

**Graphical Abstract:**

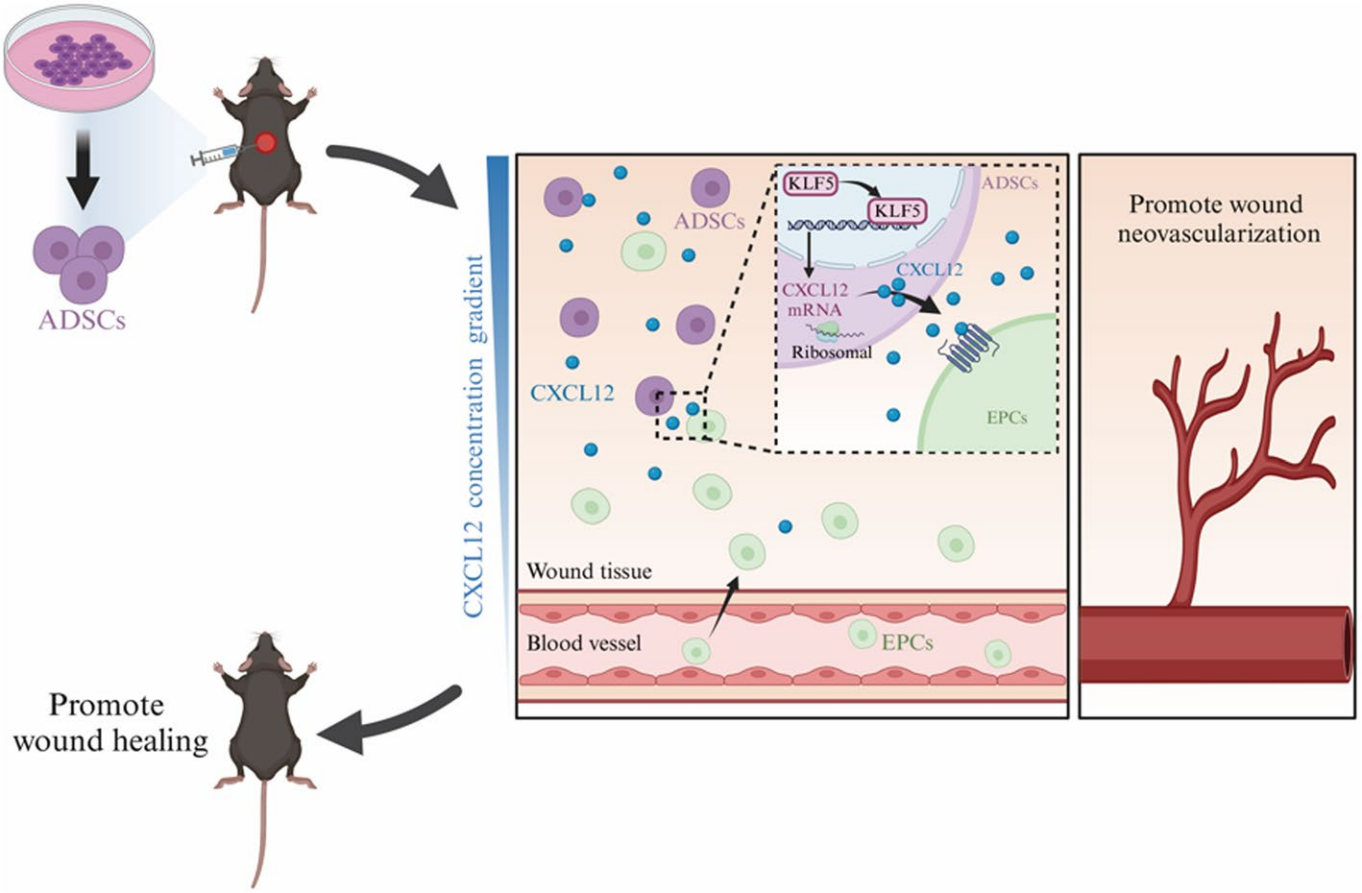

**Supplementary Information:**

The online version contains supplementary material available at 10.1186/s11658-025-00702-0.

## Background

Diabetes mellitus is a serious metabolic disease characterized by chronic hyperglycemia, with its global incidence steadily increasing. The global prevalence of diabetes is expected to reach 9.8% by 2050, affecting 1.31 billion people [1]. Among the various complications of diabetes, diabetic foot ulcers (DFUs), skin lesions on the feet that often involve the epidermis and dermis and are associated with peripheral neuropathy (PN) and/or peripheral artery disease (PAD), are particularly common, with a lifetime prevalence of 34% [2]. DFUs are also the leading cause of nontraumatic amputations owing to poor wound healing [3, 4]. Current clinical treatments for diabetic wounds primarily involve traditional methods such as debridement surgery, offloading, blood glucose management, infection control, revascularization, and conventional wound care [3]. Although new treatment modalities have emerged in recent years, their therapeutic outcomes remain unsatisfactory [5].

Research studies indicate that reduced neovascularization is a major factor contributing to the delayed healing of diabetic wounds [6, 7]. Vascular endothelial cells are essential for blood vessel formation [8], however, the hyperglycemic environment in patients with diabetes can lead to endothelial cells dysfunction. This dysfunction is driven by the formation of advanced glycation end products (AGEs), increased oxidative stress (OS) and reactive oxygen species (ROS), mitochondrial dysfunction, and activation of the polyol and hexosamine pathways, all of which disrupt angiogenic homeostasis and hinder normal wound healing [9]. Endothelial progenitor cells (EPCs) are typically mobilized and recruited to damaged ischemic tissues by local chemokines. Through processes of proliferation, migration, and differentiation, EPCs mature into endothelial cells and produce pro-angiogenic factors via paracrine signaling, contributing to the repair and maintenance of endothelial cells functions [10]. However, in the hyperglycemic conditions associated with diabetes, the proliferation, migration, differentiation, adhesion, and angiogenic capacity of EPCs are compromised, impairing vascularization at the damaged ischemic sites and leading to chronic, nonhealing wounds [9, 11]. Thus, enhancing neovascularization is critical for effective treatment of diabetic wounds.

Adipose-derived stem cells (ADSCs) have shown significant potential in regenerative medicine and tissue engineering owing to their ability to promote tissue repair and regeneration [12]. Notably, ADSCs have been demonstrated to promote angiogenesis and facilitate neovascularization, both of which are essential for tissue repair and regeneration [7, 13, 14]. Numerous studies have highlighted the positive effects of ADSCs in wound repair, noting their ability to differentiate into endothelial cells and support the regeneration of damaged blood vessels [14, 15]. Additionally, ADSCs secrete various cytokines and substantial amounts of pro-angiogenic factors via paracrine effects [16], which recruit EPCs to aid in vascular reconstruction within the injured microenvironment, thereby accelerating tissue repair and regeneration [17, 18]. Our recent study confirmed that the local injection of ADSCs into diabetic wounds in rats stimulates neovascularization and speeds up wound healing, with an associated upregulation of C-X-C motif chemokine 12 (CXCL12) expression in the wound tissues. However, the underlying mechanism remains unclear [19].

In this study, we investigated the effects of ADSCs on diabetic wound healing and EPCs functions in a hyperglycemia environment using both in vivo and in vitro diabetes models. We also confirmed the presence of complementary binding sites for Kruppel-like factor 5 (KLF5) and CXCL12 through dual-luciferase gene reporter assay. Collectively, our findings suggest that ADSCs represent a promising alternative strategy for improving diabetic wound healing.

## Methods

### Collection of human adipose-derived stem cells (hADSCs)

Adipose tissue samples were collected from patients undergoing liposuction at the Department of Plastic Surgery, First Affiliated Hospital of Fujian Medical University. All patients provided written informed consent. hADSCs were isolated from adipose tissue obtained from the thighs or abdomen, following a previously reported protocol [20]. Briefly, the aspirated adipose suspension was allowed to settle, and the upper layer of adipose tissue was collected. This tissue was then digested with an equal volume of 0.2% type I collagenase (cat. no. SCR103, Sigma-Aldrich, St. Louis, MO, USA) for 45 min at 37 °C. The enzyme activity was neutralized by adding an equal volume of mesenchymal stem cell culture medium (cat. no. MUXMD-90011, Cyagen, Suzhou, China), and the supernatant was discarded after centrifugation at 1200 rpm for 5 min. Erythrocytes were lysed by adding erythrocyte lysis buffer and incubating at room temperature for 5 min. Phosphate-buffered saline (PBS; cat. no. 10,010,023, Invitrogen, Carlsbad, CA, USA) was then added, followed by centrifugation at 1000 rpm for 5 min. The supernatant was discarded, and the remaining cells were resuspended in the mesenchymal stem cell culture medium. The cells were cultured in a 37 °C incubator with 5% CO2 to establish stable hADSCs, which were expanded through three to six passages.

### RNA extraction, RNA-sequencing, and data analysis

Total RNA was extracted from hADSCs treated under high glucose (HG, 30 mM) and low glucose (LG, 5.5 mM) conditions using the TRIzol reagent (cat. no. 15,596,026, Thermo Fisher Scientific, Waltham, MA, USA) according to the manufacturer’s protocol. Each group (HG and LG) included three cell samples. The concentration and purity of the extracted RNA were assessed using a NanoDrop spectrophotometer (Thermo Fisher Scientific), and RNA integrity was verified using an Agilent 2100 Bioanalyzer (Agilent Technologies, Santa Clara, CA, USA). Only samples with an RNA integrity number (RIN) greater than 7.0 were used for subsequent RNA sequencing. RNA sequencing (RNA-seq) libraries were prepared from 1 μg of total RNA per sample using the NEBNext Ultra II RNA Library Prep Kit for Illumina (New England Biolabs, Ipswich, MA, USA) as per the manufacturer’s guidelines. Sequencing was performed on an Illumina NovaSeq 6000 platform (Illumina, San Diego, CA, USA) to produce 150 bp paired-end reads. Raw sequencing data underwent quality control checks using FastQC (version 0.11.9). Sequencing reads were aligned to the human reference genome (GRCh38) using HISAT2 (version 2.2.1), and gene expression levels were quantified and normalized to counts per million (CPM) using FeatureCounts (version 2.0.1). All raw RNA sequencing data have been deposited in the NCBI Gene Expression Omnibus (GEO) under the BioProject accession number PRJNA1209402. The dataset can be accessed via the following link: https://dataview.ncbi.nlm.nih.gov/object/PRJNA1209402?reviewer=csf1939panbikk0miv62n71qk2.

### Identification of NRS and functional analysis

Differential gene expression analysis was performed using the limma-voom pipeline in R, identifying significant differentially expressed genes (DEGs) with an adjusted *p*-value < 0.05 and |log_2_fold change| > 0.585. A total of 4829 neovascularization-related genes were retrieved from GeneCards (https://www.genecards.org/). DEGs were intersected with these neovascularization-related genes to define the neovascularization-related signature (NRS) using the Venn package in R. Functional enrichment analysis, including the Gene Ontology (GO) and Kyoto Encyclopedia of Genes and Genomes (KEGG) pathways, was performed using the clusterProfiler package in R, focusing on biological processes (BP), cellular components (CC), and molecular functions (MF). A *p*-value < 0.05 was considered statistically significant.

### Protein–protein interaction (PPI) network analysis and screening for common bub genes

A PPI network was constructed using the STRING database (https://string-db.org/) with a medium confidence score > 0.4. The network visualization was performed using Cytoscape (Version 3.10.2). Hub genes were identified through five algorithms (Maximal Clique Centrality (MCC); Maximum Neighborhood Component (MNC); degree; closeness; and radiality) within the cytoHubba plugin of Cytoscape. The hub genes for the NRS were determined by intersecting the top ten genes from each algorithm.

### Isolation and identification of ADSCs

Male C57BL/6 mice were purchased from the Experimental Animal Center of Fujian Medical University. Mouse ADSCs were isolated from the inguinal fat tissue of these mice following a previously reported method [19]. The isolated cells were suspended in mesenchymal stem cell culture medium (Cyagen) and cultured in a 37 °C incubator with 5% CO_2_ to establish stable ADSCs over three to six passages. Flow cytometry (BD FACSCalibur, NJ, USA) was used to determine the expression of ADSCs surface markers, including CD29, CD34, CD45, and CD90, using antibodies from BioLegend (cat. nos. 102215, 128,611, 103,121, and 105,315, CA, USA). The multipotency of ADSCs was confirmed through alizarin red, oil red O, and alcian blue staining (cat. nos. MUXMD-90021, MUXMD-90031, and MUXMD-90041, all from Cyagen).

### Culture, treatment of EPCs, and transfection of ADSCs

EPCs (CP-M140) were purchased from Procell Life Science (Wuhan, China) and cultured in endothelial cell growth medium (EGM)-2 BulletKit medium (cat. no. CC-3162, Lonza, Basel, Switzerland) supplemented with 10% fetal bovine serum (FBS). To create an in vitro hyperglycemic model, EPCs were cultured in this medium with HG for 24 h. EPCs cultured in LG medium served as a negative control. HG-treated EPCs were co-cultured with 125 nM recombinant CXCL12 (rCXCL12, cat. no. 250-20A, PeproTech, NJ, USA), with or without 5 × 10^5^/ml ADSCs. Lentiviral transfection of ADSCs was performed using lentiviruses carrying short hairpin RNA (shRNA), prepared according to the manufacturer’s protocol (GenePharma, Shanghai, China). After 24 h, the medium was replaced with fresh culture medium, which was subsequently replaced with medium containing 10% FBS and 1 μg/mL puromycin (cat. no. ST551, Beyotime, Beijing, China) to select for transfected cells. Independent clones were isolated from each transfection set through limiting dilution analysis and screened for KLF5 or CXCL12 knockdown.

### Enzyme-linked immunosorbent assay (ELISA)

The concentration of secreted CXCL12 in the ADSCs culture medium were measured with the DuoSet ELISA kit (cat. DY460, R&D Systems, USA) following the manufacturer’s instructions. Briefly, 96-well plates were coated with CXCL12 antibody, and 100 μl of standards or samples were added to each well, followed by a 2-h incubation at room temperature. After washing each well three times, 100 μl of detection antibody was added, and the plates were incubated for another 2 h at room temperature. Following three additional washes, 100 μl of substrate solution was added and incubated for 20 min at room temperature in the dark. The absorbance was measured at 450 nm using a multifunctional microplate reader (BioTek Synergy, VT, USA).

### Western blot analysis

Total protein was extracted and quantified using a total protein extraction kit and a BCA protein assay kit (cat. nos. P0013G and P0010, both from Beyotime, Beijing, China) following the manufacturer’s instructions. Proteins were separated using sodium dodecyl-sulfate polyacrylamide gel electrophoresis (SDS-PAGE, cat. no. PG113, EpiZyme, Shanghai, China) and transferred onto polyvinylidene fluoride (PVDF) membranes (Millipore, USA). The membranes were blocked at room temperature for 30 min with protein-free rapid blocking buffer (cat. no. PS108P, EpiZyme). Primary antibodies were added, and the membranes were incubated overnight at 4 °C with gentle shaking. The primary antibodies used were anti-glyceraldehyde-3-phosphate dehydrogenase (GAPDH) (cat. no. 10494-1-AP, Proteintech, China), anti-CXCL12 (cat. no. ab9797, Abcam, UK), and anti-KLF5 (cat. no. ab137676, Abcam). After washing the membranes three times with TBST, secondary antibodies (Abcam) were added for 2 h at room temperature. Western blot bands were visualized using the Odyssey Infrared Imaging System (LI-COR Biosciences, USA), and band densities were quantified using ImageJ software, normalized to GAPDH expression, and compared with control group values.

### Reverse transcription quantitative polymerase chain reaction (RT-qPCR)

Total RNA was extracted using the RNA Easy Fast Tissue/Cell Kit (cat. no. DP451, TIANGEN, China). RNA was reverse transcribed into cDNA using the HiScript II qRT SuperMix kit (cat. no. R223, Vazyme Biotech Co., Ltd). Quantitative PCR was performed using the SYBR Green PCR kit (cat. no. Q221, Vazyme Biotech Co., Ltd). Gene expression was quantified using the 2^−ΔΔCT^ method with GAPDH as a reference. Primer sequences were as follows: CXCL12 (forward: TGCATCAGTGACGGTAAACCA; reverse: TTCTTCAGCCGTGCAACAATC) and GAPDH (forward: AGTGCCAGCCTCGTCTCATA; reverse: GATGGTGATGGGTTTCCCGT).

### Cell proliferation assay

Cell proliferation was assessed using the 5-ethynyl-2′-deoxyuridine (EdU) Cell Proliferation Detection Kit (cat. no. C0071, Beyotime). EPCs (2 × 10^4^ cells/well) were seeded in 24-well plates and cultured for 24 h under different treatments. After a 4-h incubation with EdU, cells were fixed with 4% paraformaldehyde and permeabilized with 0.3% Triton X-100. EdU-positive cells were stained with Hoechst 33,342 and visualized using a fluorescence microscope (ZEISS, Germany). Results were quantified with ImageJ software.

### Cell migration assay

EPCs (2 × 10^4^ cells/well) were seeded into the upper chamber of Transwell inserts (Corning, USA) with 500 µL of culture medium in the lower chamber. After incubation at 37 °C for 24 h, migrated cells were fixed with 4% paraformaldehyde and stained with 0.1% crystal violet. Migrated cells were imaged with a microscope (Olympus, Japan) and quantified using ImageJ software.

### Tube formation assay

The angiogenic capability of EPCs was evaluated using a Matrigel tube formation assay. Wells of a 24-well plate were coated with 100 µL of growth factor-reduced Matrigel (cat. no. 356230, Corning). EPCs were co-cultured with rCXCL12 (1.25 µg/mL) and pretreated ADSCs in 200 µL EGM-2 medium at a density of 5 × 10^4^ cells/well. After 12 h of incubation at 37 °C with 5% CO2, tube formation was visualized using an inverted microscope (Olympus).

### Diabetic wound model and wound healing assessment

Diabetes was induced in male C57BL/6 mice through intraperitoneal injection of streptozotocin (STZ, 1%, 60 mg/kg; cat. no. S0130, Sigma-Aldrich, St. Louis, MO, USA) for 5 days. Hyperglycemia (blood glucose > 16.7 mmol/L) was confirmed one week after the final injection. The mice were anesthetized with an intraperitoneal injection of sodium pentobarbital (40 mg/kg). Full-thickness circular skin wounds (0.6 cm diameter) were created on the dorsum of each anesthetized mouse. A silicone ring was sutured around the wound to prevent contracture. The mice were randomly assigned to six groups on the basis of different treatments: control, diabetes mellitus (DM), shNC ADSCs, sh-KLF5 ADSCs, sh-CXCL12 ADSCs, and rCXCL12. After establishing the skin wound model, 100 µL of PBS, 100 µL of PBS-diluted ADSCs (1 × 10^6^) or rCXCL12 (25 µg/kg) was injected into the wound via local subcutaneous injection. Wound size was measured on days 0, 1, 3, 7, 10, 14, and 21, and digital images were captured. Wound tissues were harvested for analysis after euthanasia.

### Histological observation

Wound tissues collected on days 1, 3, 7, 10, 14, and 21 post-surgery were fixed in 4% paraformaldehyde overnight and dehydrated in an ethanol-xylene series before embedding in paraffin. Sections (4–6 µm) were stained with hematoxylin and eosin (HE) and Masson’s trichrome (cat. nos. R32933 and R20379, Yuanye, Shanghai, China) for morphological analysis under a microscope (Olympus).

### Immunohistochemistry and immunofluorescence

Sections of wound tissues from days 1, 3, 7, 10, 14, and 21 were stained with anti-CD31(1:100, cat. no. AF3628, R&D Systems) and anti-α-smooth muscle actin (α-SMA) antibodies (1:200, cat. no. 14,395-1-AP, Proteintech) for immunofluorescence. After overnight incubation with primary antibodies at 4 °C, sections were incubated with secondary antibodies at 37 °C for 1 h. The sections were subsequently stained with 4′,6-diamidino-2-phenylindole (DAPI, Invitrogen, Carlsbad, CA) and examined under a Zeiss LSM 780 confocal fluorescence microscope (ZEISS).

### Luciferase reporter gene assay

For luciferase activity assays, cells were co-transfected with a KLF5 luciferase reporter plasmid and a *Renilla* luciferase reporter vector (GPL4) per well using Lipofectamine™ 2000 (cat. no. 11668019, Invitrogen, USA) for 24 h. Cells were then harvested for luciferase activity analysis using the dual-luciferase reporter gene assay system (cat. no. RG027, Beyotime, Haimen, China). Luminescence was measured with a GloMax^®^ 96 Microplate Luminometer with Dual Injectors (Promega, Madison, USA). The firefly luciferase luminescence activity was normalized to the control *Renilla* luciferase activity.

### Statistical analysis

All data were analyzed using Prism 9 software (GraphPad Software v9.0, CA, USA). Experiments were performed in triplicate, and results are expressed as mean ± SEM. One-way analysis of variance (ANOVA) followed by Tukey’s post hoc test was used for multiple comparisons. Statistical significance was set at *p*-value < 0.05 (*p < 0.05, **p < 0.01, ***p < 0.001, and ****p < 0.0001).

## Results

### Potential mechanisms of ADSCs in promoting wound neovascularization and accelerate repair in diabetic mice

**Fig. 1:**
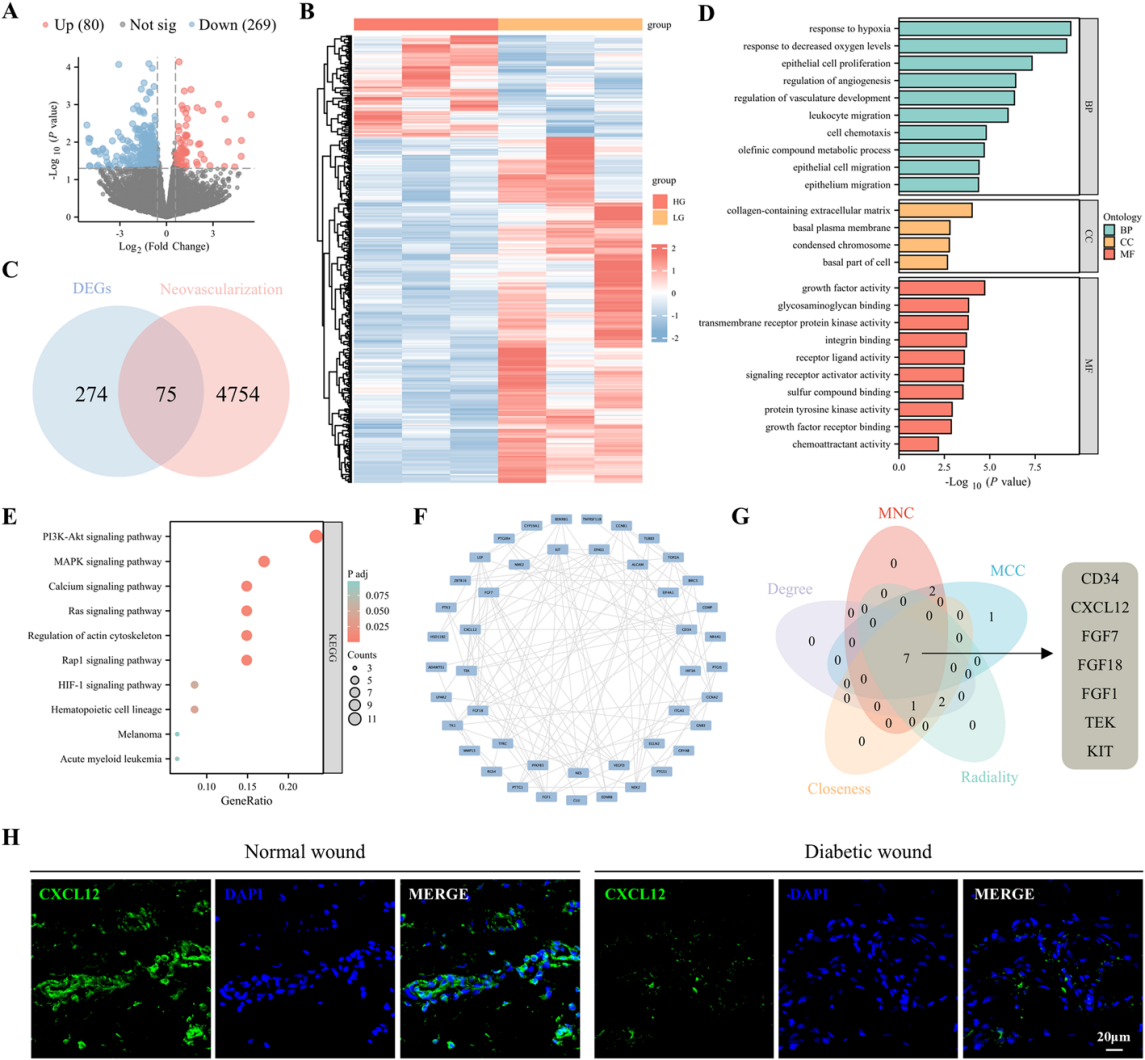
Bioinformatics analysis of potential mechanisms of ADSCs affecting neovascularization in high glucose (HG). **A** Volcano plot of differentially expressed mRNAs depicting upregulated and downregulated genes. **B** Heat map showing differentially expressed genes in ADSCs treated with LG or HG. **C** Venn diagram showing 75 shared genes between DEGs and the neovascularization phenotype gene set. **D** Results of GO analysis. **E** Results of KEGG analysis. **F** The PPI network of 75 shared genes. **G** Seven common hub genes were identified by five algorithms (MCC, MNC, degree, closeness, and radiality) of the cytoHubba plugin. **H** Representative images of tissue sections from patients with diabetic ulcers immunostained for CXCL12 (green). GO, Gene Ontology; KEGG, Kyoto Encyclopedia of Genes and Genomes; BP, biological process; CC, cellular component; MF, molecular function; FGF7, fibroblast growth factor 7; FGF18, fibroblast growth factor 18; FGF1, fibroblast growth factor 1; TEK, TEK receptor tyrosine kinase; KIT, proto-oncogenic receptor tyrosine kinase

To explore the potential mechanisms by which adipose-derived stem cells (ADSCs) promote neovascularization in diabetic wound repair, we analyzed changes in mRNA expression 48 h after treating hADSCs under HG and LG conditions using high-throughput transcriptome sequencing. Genes with a *p*-value < 0.05 and |log_2_fold change| > 0.58 were considered significantly DEGs. A total of 80 upregulated and 269 downregulated mRNAs were identified, as depicted in the volcano plot and heat map (Fig. 1A, B). The neovascularization-related genes containing 4830 genes were retrieved from GeneCards. By intersecting the 349 DEGs with neovascularization-related genes, 75 shared genes were identified to construct NRS (Fig. 1C).

GO enrichment analysis (Fig. 1D) and KEGG pathway analysis (Fig. 1E) were conducted to examine the association of NRS with specific on biological processes (BP), cellular components (CC), and molecular functions (MF) and pathways. The analysis revealed that NRS were involved in BP related to the response to hypoxia and decreased oxygen levels. CC and MF were associated with the collagen-containing extracellular matrix and were involved in growth factor activity, glycosaminoglycan binding, and transmembrane receptor protein kinase activity. The KEGG pathway analysis indicated significant enrichment in pathways such as phosphatidylinositol 3-kinase-protein kinase B (PI3K-Akt), mitogen-activated protein kinase (MAPK), calcium signaling, Ras signaling, regulation of actin cytoskeleton, and Rap1 signaling pathway.

To explore protein interactions, we constructed a PPI network of NRS using the STRING database, resulting in a network of 46 nodes and 116 edges, visualized using Cytoscape (Fig. 1F). The top ten hub genes were screened using five algorithms (MCC, MNC, degree, closeness, and radiality) in the cytoHubba plugin. Seven hub genes (CD34, CXCL12, FGF7, FGF18, FGF1, TEK, and KIT) were identified by taking the intersection of the top ten genes from all algorithms (Fig. 1G). Immunofluorescence staining revealed that CXCL12 expression was significantly reduced in the wound tissue of patients with diabetic ulcers compared with normal skin (Fig. 1H). CXCL12, in particular, was selected for further preliminary studies owing to its potential significance.

### Characterization of ADSCs and verification of CXCL12 knockdown

**Fig. 2:**
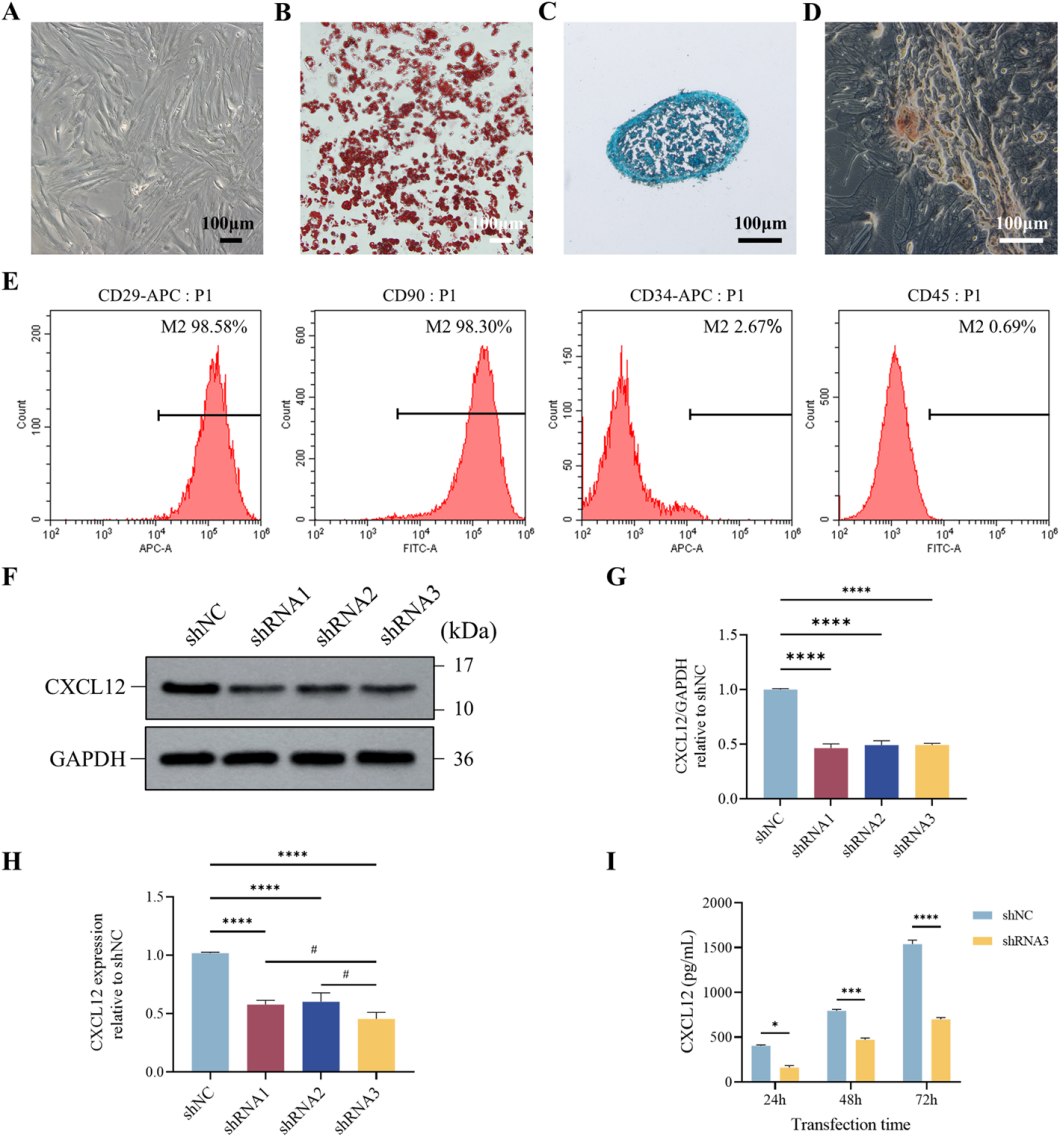
Characterization of ADSCs and verification of CXCL12 knockdown. **A** Normal cultured ADSCs under a microscope, scale bar = 100 μm; **B** ADSCs after adipogenic differentiation, scale bar = 100 μm. **C** ADSCs after chondrogenic differentiation, scale bar = 100 μm. **D** ADSCs after osteogenic differentiation, scale bar = 100 μm. **E** Flow cytometry analysis of ADSC surface markers: CD45, CD34, CD29, and CD90. **F** Western blot analysis of CXCL12 protein expression in each group of ADSCs. **G** Western blot quantitative analysis of CXCL12 expression level. **H** RT-qPCR detection of CXCL12 mRNA expression in each group of ADSCs. **I** ELISA detection of CXCL12 secretion in the culture supernatant of ADSCs. All experiments were performed in triplicate and were repeated three times to confirm the findings (*p < 0.05). Data are presented as mean ± standard deviation (SD). *p < 0.05, ***p < 0.001, ****p < 0.0001 versus shNC; ^#^*p* < 0.05 versus shRNA3

ADSCs were observed to grow well in a monolayer with a fibroblast-like morphology (Fig. 2A). Their differentiation potential was confirmed by positive staining for adipocytes with oil red O (Fig. 2B), chondrocytes with alcian blue (Fig. 2C), and osteoblasts with alizarin red (Fig. 2D). Flow cytometry analysis demonstrated that ADSCs expressed mesenchymal stem cell markers CD29 (98.58%) and CD90 (98.30%) and showed minimal expression of hematopoietic cell markers CD34 (2.67%) and CD45 (0.69%), confirming successful isolation of ADSCs (Fig. 2E). To maintain cell uniformity during experiments, ADSCs were used between passages three and six (P3–P6).

To further investigate the biological effects of CXCL12, ADSCs were transfected with lentiviral shRNA targeting CXCL12 (sh-CXCL12). The knockdown efficiency was verified by RT-qPCR, western blot, and ELISA, which demonstrated significant reductions in both mRNA and protein levels of CXCL12 in ADSCs (Fig. 2F–I).

### ADSCs promote wound healing and neovascularization in diabetic wounds through CXCL12

**Fig. 3:**
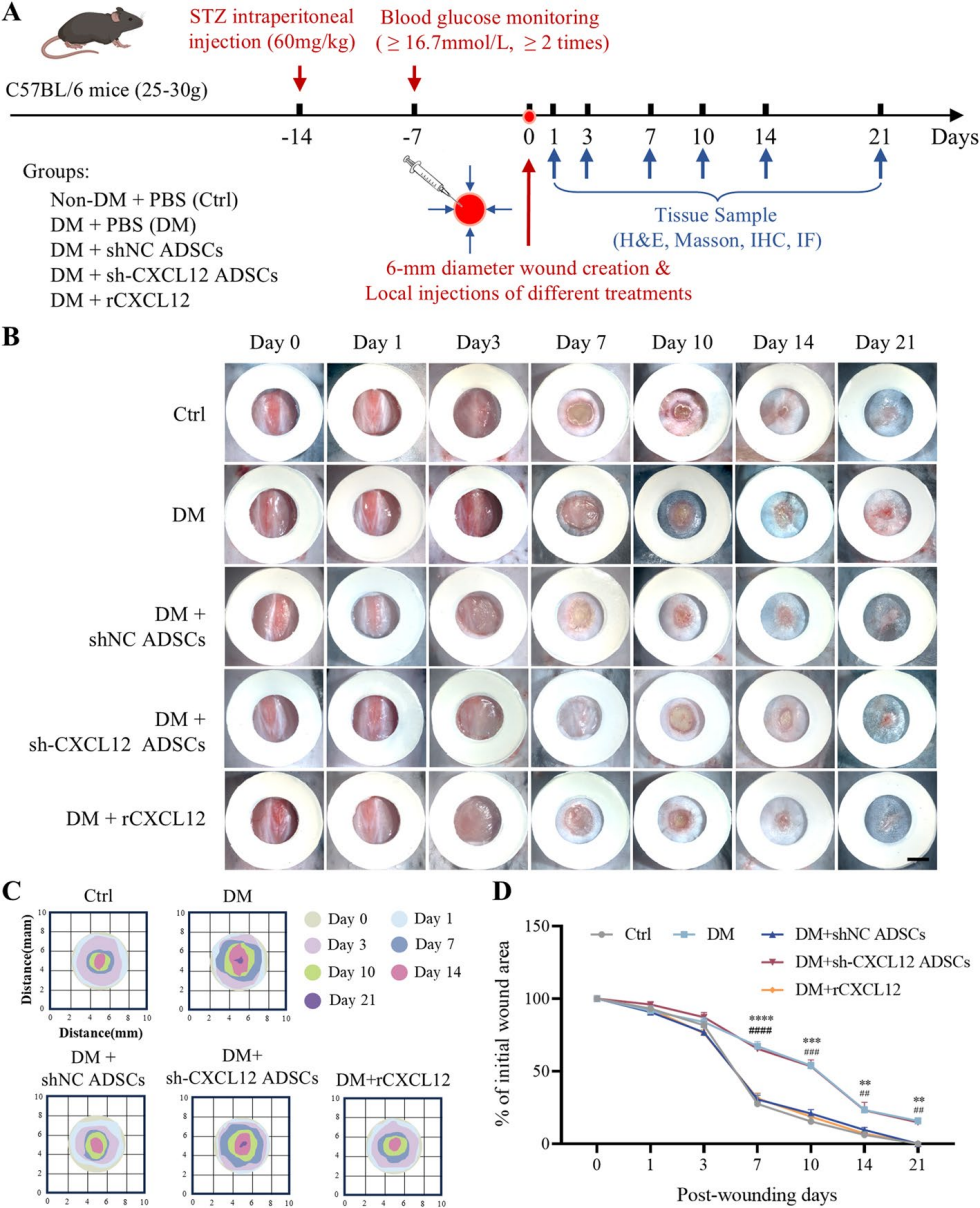
ADSCs transplantation promotes diabetic wound healing via CXCL12. **A** Generating a full-thickness skin defect model and treatment protocol. **B** Representative images of wound healing process in mice in each group. Scale bar = 5 mm. (**C**) Schematic diagram of the wound managed by different treatments in 21 days. **D** The wound closer percentage was evaluated post-wounding. Data represent mean ± SD (*n* = 21 animals per group), **p < 0.01, ***p < 0.001, ****p < 0.0001 versus control; ^#^*p* < 0.05, ^###^*p* < 0.001, ^####^*p* < 0.0001 versus DM

**Fig. 4:**
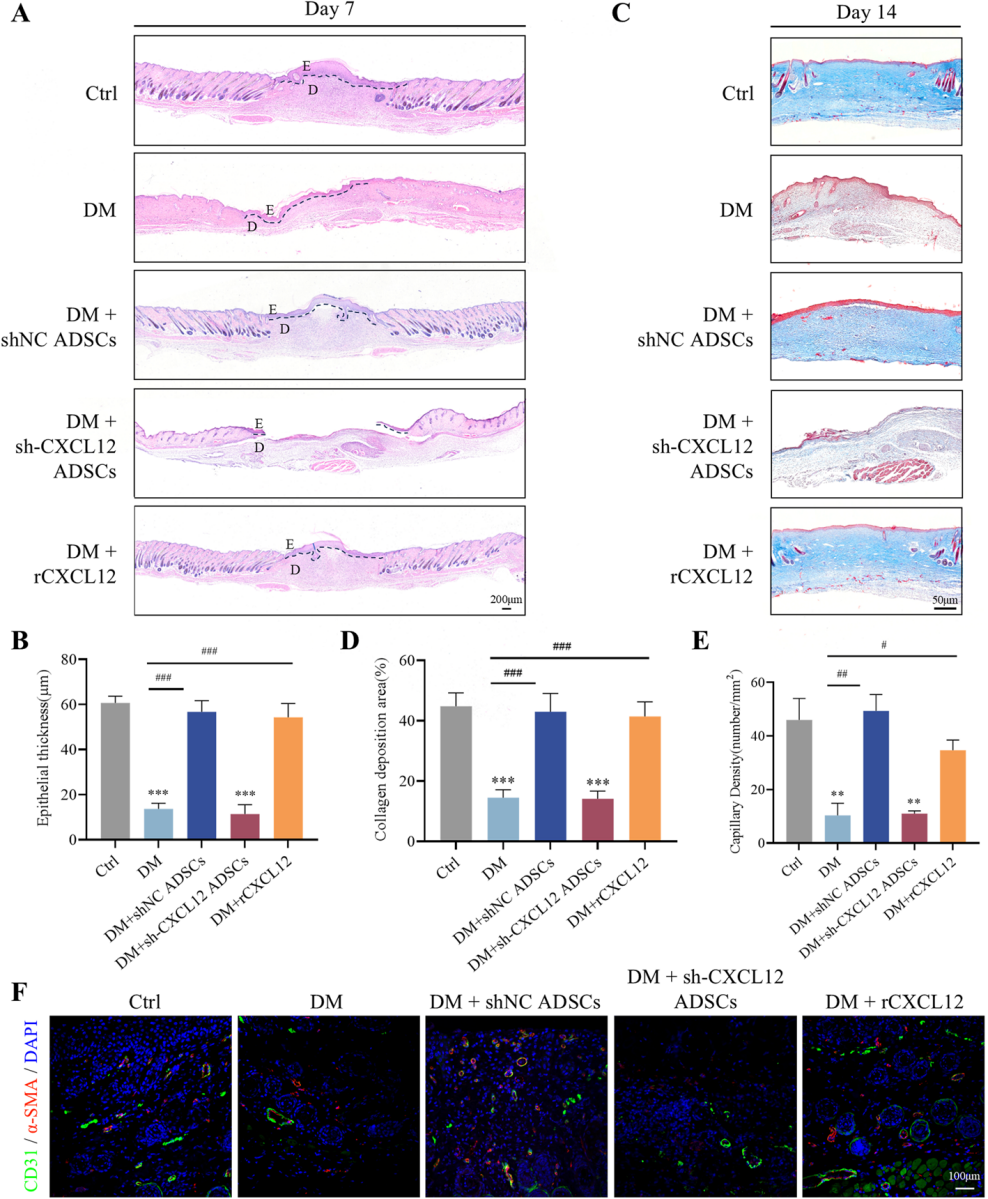
Evaluation of wound re-epithelialization and blood vessel formation. **A** The HE staining of the skin wound of diabetic mice on day 7 post-wounding was constructed; the dashed line is the boundary between epidermis and dermis; E is the epidermis, and D is the dermis. Scale bar = 200 μm. **B** Quantitative analysis of epithelialization in wound tissue 7 days after different treatments. **C** Evaluation of collagen maturity by staining wounds with Masson’s trichrome following different treatments 2 weeks post-wounding. Scale bar = 50 μm. **D** Quantitative analysis of collagen in wound tissue 14 days after different treatments. **E** Representative images of tissue sections from patients with diabetic ulcers immunostained for CD31 (green) and α-SMA (red). scale = 100 μm. **F** Quantitative analysis of capillary density in wound tissue after different treatments. All experiments were performed in triplicate and were repeated three times to confirm the findings (*p < 0.05). Data represent mean ± SD, **p < 0.01, ***p < 0.001 versus control; ^#^*p* < 0.05, ^##^*p* < 0.01, ^###^*p* < 0.001 versus DM

A diabetic mouse model was successfully established with a circular full-thickness skin defect wound created on the dorsal side of each mouse, as shown in Fig. 3A. The results showed that wound healing was significantly accelerated in mice treated with either shNC ADSCs or recombinant CXCL12 (rCXCL12) compared with the control group. However, downregulation of CXCL12 expression inhibited the therapeutic effect of ADSCs on wound healing (Fig. 3B–D). HE staining (Fig. 4A, B) and Masson’s trichrome staining (Fig. 4C, D) demonstrated that treatment with shNC ADSCs or rCXCL12 significantly increased the epithelial thickness of the traumatized tissues in diabetic mice and enhanced collagen deposition in diabetic mice. In contrast, the group treated with sh-CXCL12 ADSCs exhibited significantly reduced epithelialization and disorganized collagen arrangement in the wound bed. Immunostaining for CD31 and α-SMA (Fig. 4E, F) indicated enhanced angiogenesis in the shNC ADSCs and rCXCL12 groups, while CXCL12 silencing reversed the angiogenic promotion by shNC ADSCs. These results suggest that ADSCs significantly stimulate wound healing, improve re-epithelialization, increase collagen deposition, and promote neovascularization in diabetic wounds, primarily through CXCL12.

### KLF5 as an upstream transcription factor of CXCL12

**Fig. 5:**
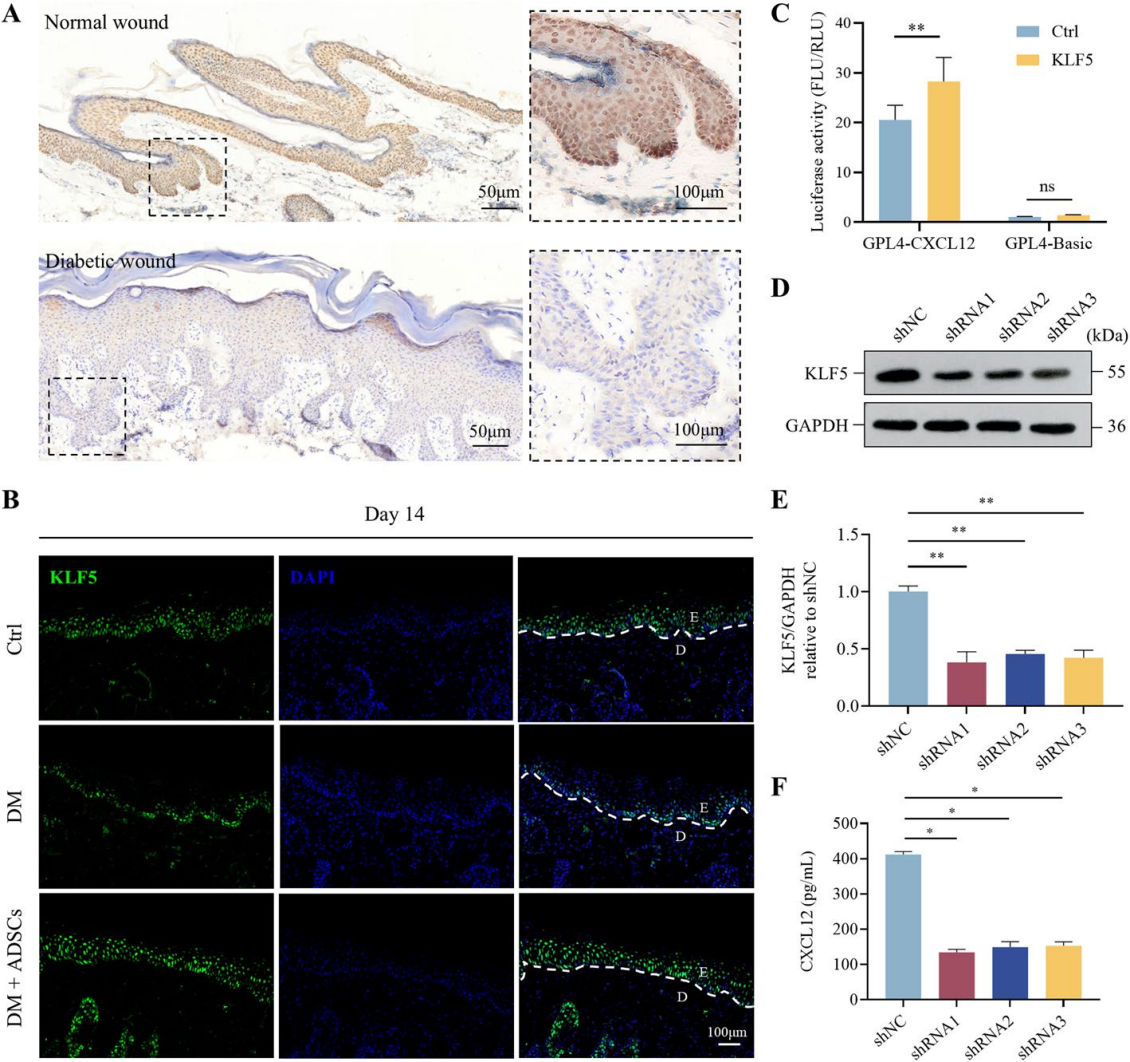
Expression of KLF5 in wound tissue and the verification of the targeted regulation relationship between KLF5 and CXCL12. **A** Histological analysis of KLF5 in wound tissue in patients with diabetic ulcers and diabetic mice. × 100, Scale bar = 50 μm; ×200, Scale bar = 100 μm. **B** Immunofluorescence diagram of KLF5 in the wound tissue of diabetic mice on day 14 post-wounding; dashed line is the boundary between epidermis and dermis; E is the epidermis and D is the dermis. Scale bar = 50 μm. **C** Targeted modulation was measured by luciferase reporter gene assays. The ratios of Firefly luciferase (FLU) activity to *Renilla* luciferase (RLU) activity are displayed. **D** Western blot analysis of KLF5 protein expression in each group of ADSCs. **E** Western blot quantitative analysis of KLF5 expression level. **F** ELISA detection of CXCL12 secretion in the culture supernatant of ADSCs. All experiments were performed in triplicate and were repeated three times to confirm the findings (*p < 0.05). Data represent mean ± SD, *p < 0.05, ** *p* < 0.01, ns, not significant

To elucidate the mechanism by which CXCL12 in ADSCs promotes diabetic wound healing, we investigated its upstream regulatory molecules. Potential upstream transcription factors of CXCL12 were predicted using the JASPAR database, revealing several candidates: KLF5, NFKB1, CREB1, RELA, STAT3, E2F1, PPARG, TFAP2C, and TFAP2A. We focused on KLF5 owing to its potential involvement in diabetic wound healing. Immunohistochemical staining confirmed that KLF5 expression was low in the wound tissues of patients with diabetic ulcers and diabetic mice (Fig. 5A, B). Notably, KLF5 expression increased significantly in diabetic mouse wound tissues following ADSC treatment (Fig. 5B).

Using the JASPAR database (http://jaspar.genereg.net/), we screened the promoter region of CXCL12 and identified 79 potential KLF5 binding sites within the 1399 bp region upstream of the CXCL12 5′ UTR. To determine whether KLF5 regulates CXCL12 transcription via promoter binding, a dual-luciferase reporter assay was performed. Overexpression of KLF5 resulted in increased luciferase activity, indicating activation of the CXCL12 promoter (Fig. 5C). Stable KLF5 knockdown and control ADSCs were generated by transfecting cells with sh-KLF5 and shNC lentiviruses, respectively. Western blot analysis confirmed the knockdown efficiency (Fig. 5D, E). ELISA measurements showed that CXCL12 expression was significantly reduced in KLF5 knockdown ADSCs compared with controls (Fig. 5F). These results suggest that KLF5 directly binds to the promoter of CXCL12 and activates its secretion.

### KLF5 downregulation impairs healing in diabetic wounds

**Fig. 6:**
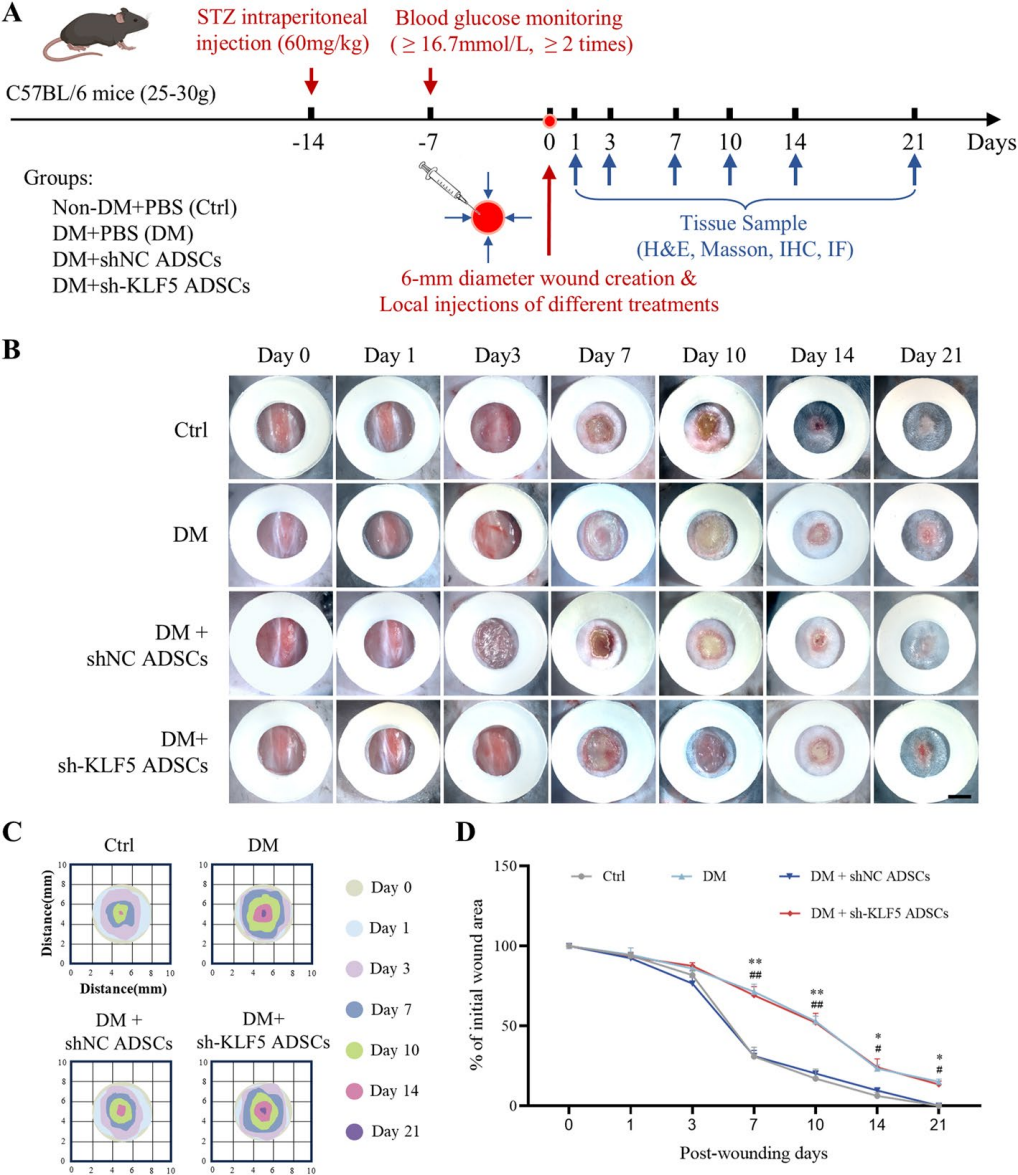
Downregulation of KLF5 delays wound healing in diabetic wound. **A** Generating a full-thickness skin defect model and treatment protocol. **B** Representative images of wound healing process in mice in each group. Scale bar = 5 mm. **C** Schematic diagram of the wound managed by different treatments in 21 days. **D** The wound closer percentage was evaluated post-wounding. All experiments were performed in triplicate and were repeated three times to confirm the findings (*p < 0.05). Data represent mean ± SD (*n* = 21 animals per group), *p < 0.05, **p < 0.01 versus control; ^#^*p* < 0.05, ^##^*p* < 0.01 versus DM

**Fig. 7:**
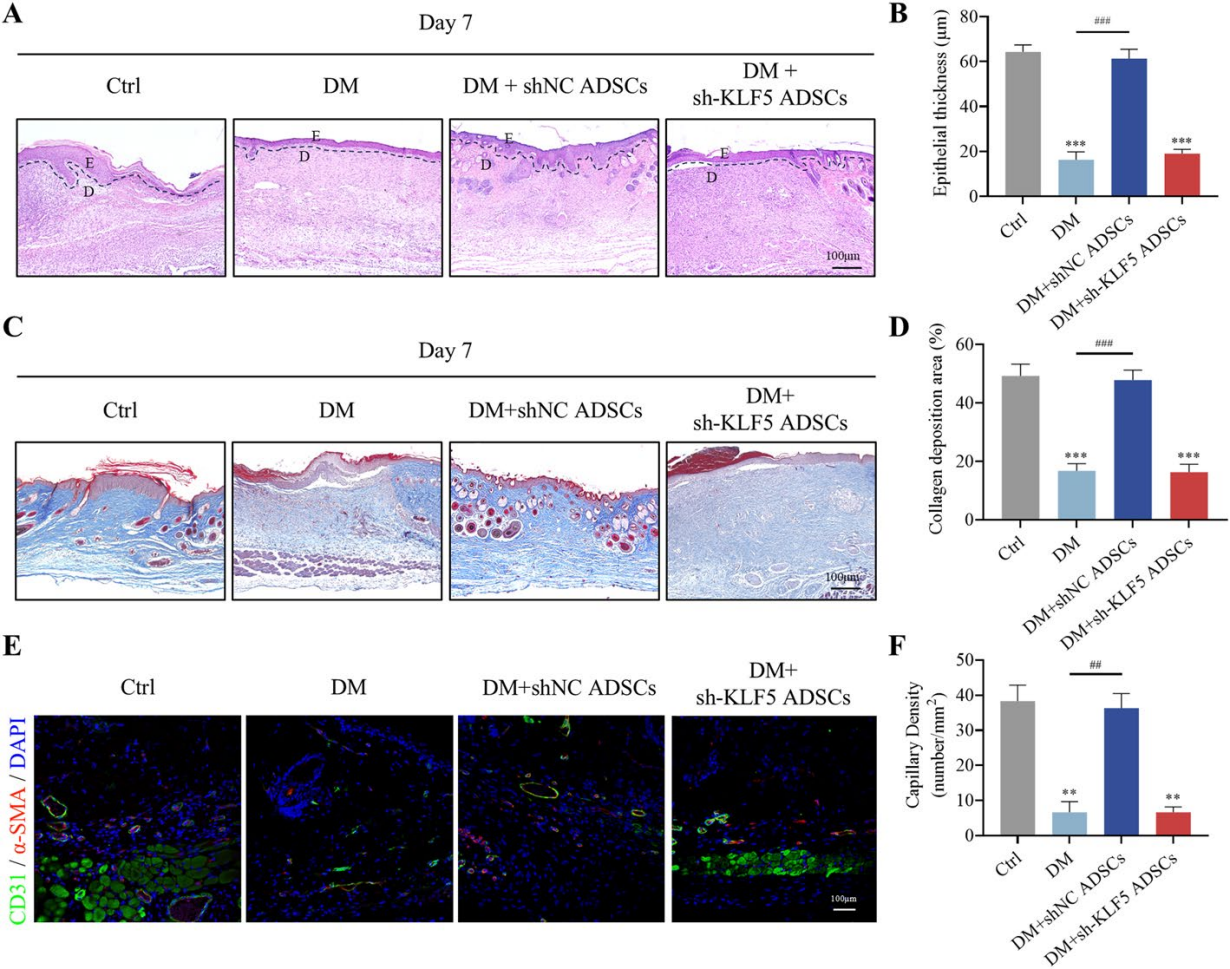
Downregulation of KLF5 inhibits re-epithelialization and blood vessel formation in diabetic wound. **A** The HE staining of the skin wound of diabetic mice on day 7 post-wounding was constructed; the dashed line is the boundary between epidermis and dermis; E is the epidermis, and D is the dermis. Scale bar = 100 μm. **B** Quantitative analysis of epithelialization in wound tissue 7 days after different treatments. **C** Evaluation of collagen maturity by staining wounds with Masson’s trichrome following different treatments 2 weeks post-wounding. Scale bar = 100 μm. **D** Quantitative analysis of collagen in wound tissue 14 days after different treatments. **E** Representative images of tissue sections from patients with diabetic ulcers immunostained for CD31 (green) and α-SMA (red). Scale bar = 100 μm. **F** Quantitative analysis of capillary density in wound tissue after different treatments. All experiments were performed in triplicate and were repeated three times to confirm the findings (*p < 0.05). Data represent mean ± SD, **p < 0.01, ***p < 0.001 versus control; ^##^*p* < 0.01, ^###^*p* < 0.001 versus DM

To assess the role of KLF5 in the therapeutic efficacy of ADSCs, we established a diabetic mouse wound model and applied different treatment regimens (Fig. 6A). The wound healing rate was significantly lower in the group treated with sh-KLF5 ADSCs compared with the shNC ADSCs group, indicating that KLF5 knockdown inhibited ADSC-mediated wound healing in diabetic mice (Fig. 6B–D). HE staining revealed a marked reduction of wound epithelialization in the sh-KLF5 ADSCs group, similar to the DM group (Fig. 7A, B). Masson’s trichrome staining showed decreased and disorganized collagen deposition in the sh-KLF5 ADSCs group compared with the shNC ADSCs group (Fig. 7C, D). Additionally, immunofluorescence staining for CD31 and α-SMA confirmed significantly reduced neovascularization in the wound area of the sh-KLF5 ADSCs group relative to the shNC ADSCs group (Fig. 7E, F). These findings underscore the critical role of KLF5 in the therapeutic effects of ADSCs on diabetic wound healing.

### ADSCs modulate the KLF5/CXCL12 pathway to enhance EPCs proliferation, migration, and vasculogenic capacity

**Fig. 8:**
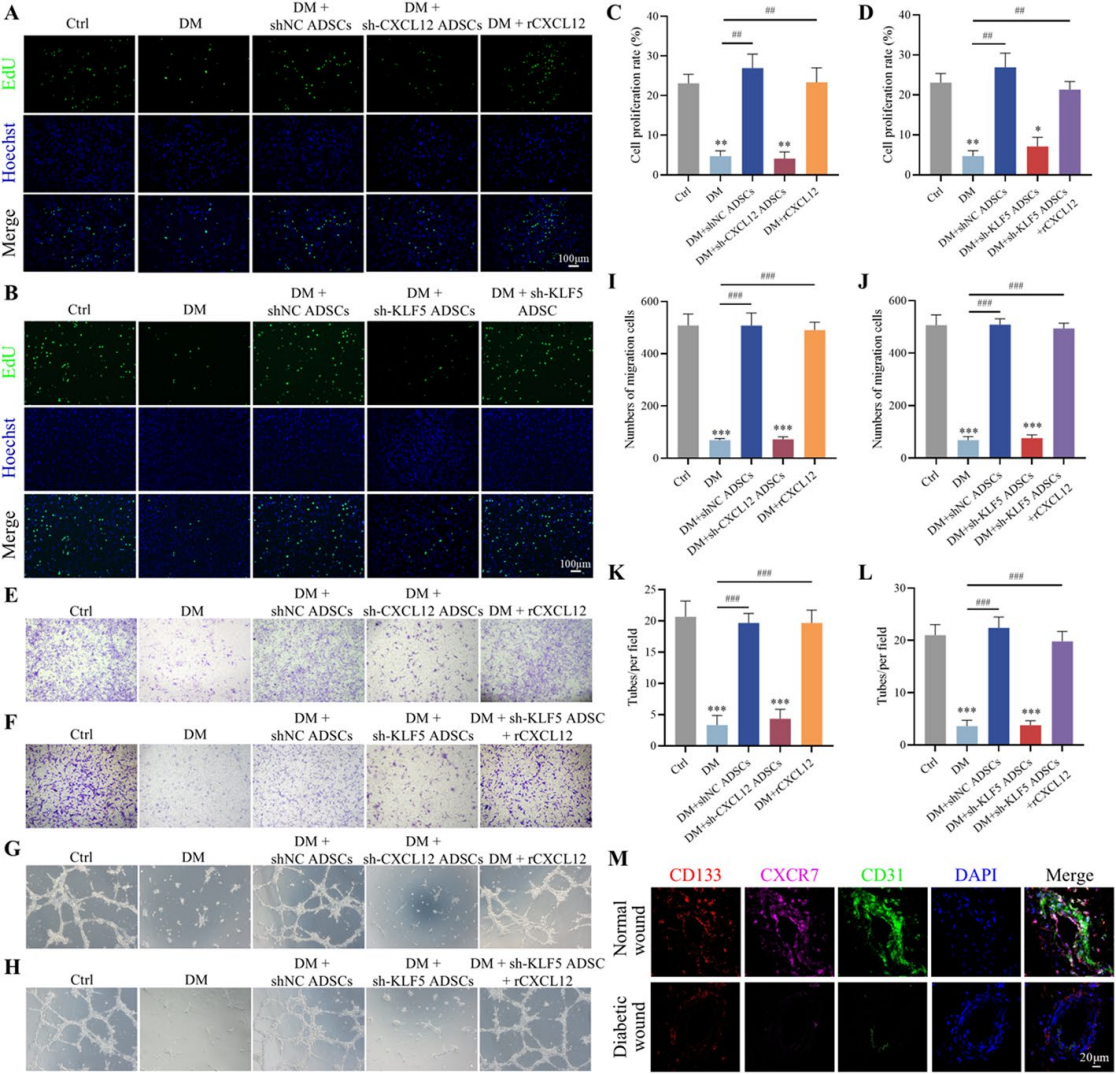
ADSCs regulate KLF5 / CXCL12 to affect the proliferation, migration and vasculogenic capacity of EPCs. **A**-**D** EdU assay measures the cell proliferation of EPCs. Scale bar = 100 μm. **E**–**F**, **I**-**J** Transwell assay measures the cell migration of EPCs. Scale bar = 100 μm. **G**-**H**, **K**-**L** Tube formation assay measures the vasculogenic capacity of EPCs. Scale bar = 100 μm. (**M** Representative images of tissue sections from patients with diabetic ulcers immunostained for CD133 (red), CXCR7 (purple), and CD31 (green). All experiments were performed in triplicate and were repeated three times to confirm the findings (*p < 0.05). Data represent mean ± SD, *p < 0.05, **p < 0.01, ***p < 0.001 versus control; ^##^*p* < 0.01, ^###^*p* < 0.001 versus DM

EPCs are crucial for neovascularization, yet their proliferation, migration, and vasculogenic capacity are compromised under HG medium, contributing to delayed wound healing in diabetic conditions. To investigate the effect of ADSCs on the cellular functions of EPCs, we established an in vitro HG model. EdU assays demonstrated that co-culturing EPCs with shNC ADSCs or rCXCL12 for 24 h significantly improved their proliferation capacity compared with the DM group. This enhancement was suppressed when KLF5 or CXCL12 expression was downregulated in ADSCs. Notably, adding rCXCL12 after KLF5 knockdown in ADSCs restored EPCs proliferation ability (Fig. 8A–D). Similar trends were observed for EPCs migration and vasculogenic capacity. The Transwell migration and tube formation assays revealed that rCXCL12 significantly increased EPCs migration and vasculogenic capacity when co-cultured with sh-KLF5 ADSCs (Fig. 8E–L). Immunofluorescence staining revealed that the expression of CD133, a surface marker of EPCs, was slightly lower in patients with diabetes compared with normal controls. In contrast, the levels of C-X-C motif chemokine receptor 7 (CXCR7; a receptor for CXCL12) and the endothelial cell marker CD31 were significantly reduced in wound tissues from patients with diabetic ulcers compared with normal skin. In the normal control group, CXCR7-positive cells were found to be highly co-localized with CD133-positive cells (Fig. 8M). This reduction in CXCR7 and CD31 suggests a decreased number of endothelial cells in diabetic ulcer wound tissues, potentially hindering EPC differentiation and impairing the neovascularization process. The diminished expression of CXCR7 on the surface of EPCs in patients with diabetes may contribute to these effects.

## Discussion

Normal wound healing progresses through hemostatic, inflammatory, proliferative, and remodeling phases. Any disruption in these phases can hinder the healing process, potentially leading to chronic nonhealing wounds, such as diabetic wounds [21, 22]. Current conventional clinical treatments have significant limitations in addressing these wounds, highlighting the urgent need for innovative and more effective treatment strategies [22].

Neovascularization, which includes both angiogenesis and vasculogenesis, is essential for proper wound healing and successful tissue repair [7, 23]. Angiogenesis involves the formation of new blood vessels from preexisting ones. It includes the sprouting, splitting, or elongation of existing endothelial cells to form new branches of blood vessels. Vasculogenesis refers to the formation of new blood vessels from endothelial progenitor cells (EPCs) or angioblasts, which are precursor cells. This process is crucial during embryonic development for the formation of the primary vascular network [24, 25]. The primary challenge in diabetic wound healing is insufficient neovascularization, which delays the repair process. [8, 9, 22, 26]. Therefore, enhancing neovascularization could be a critical step in preventing and treating DFUs.

In our previous research, we found that CXCL12 expression was significantly reduced in diabetic wounds. However, local injection of adipose-derived stem cells (ADSCs) increased both neovascularization and CXCL12 expression. [19]. CXCL12 is a major chemokine responsible for recruiting EPCs to the wound microenvironment, a key process in wound healing. [27, 28].Our results indicated that knocking down CXCL12 in ADSCs using shRNA significantly impaired their therapeutic effects on diabetic wounds, leading to reduced re-epithelialization, collagen alignment, and neovascularization. These findings suggest that ADSC-mediated wound healing is closely tied to CXCL12 secretion, a conclusion supported by numerous studies [29–32].

However, clinical application of exogenous recombinant CXCL12 protein is limited by its short half-life and rapid degradation [33]. In contrast, ADSCs, as natural carriers of CXCL12, secrete higher levels of the chemokine, making them a promising therapeutic option. Although, numerous clinical studies have consistently shown that ADSCs are not retained long-term at the recipient site after transplantation [34, 35], they are supposed to secrete essential proteins quickly, minimizing the risk of adverse reactions while enhancing therapeutic efficacy. This led us to explore how CXCL12 secretion is regulated by ADSCs.

We identified KLF5 as an upstream transcription factor regulating CXCL12. KLF5, a zinc finger transcription factor, plays a critical role in vascular homeostasis and angiogenesis [36, 37]. Research has demonstrated that the transcription factor FOXO1 enhances KLF5 transcription by binding to its promoter in cardiac myocytes isolated from patients with diabetic cardiomyopathy, thereby modulating KLF5 expression and influencing cardiac function [38], while the role of KLF5 in diabetic wound healing remains unexplored. Previous studies have found that the formation of new blood vessels is reduced in KLF5^±^ mice, and both dermal microvascular endothelial cells and EPCs derived from these KLF5^±^ mice exhibit functional defects in vascular formation [39]. Additionally, KLF5 expression is reduced in prostate cancer, leading to the inhibition of angiogenesis [40], whereas in breast cancer, KLF5 promotes angiogenesis by binding to the promoters of tumor necrosis factor (TNF)α-induced protein 2 (TNFAIP2) [41]. However, its role in diabetic wound healing has not been previously explored.

Our findings revealed that KLF5 expression is significantly lower in diabetic ulcer tissues from both patients and mice but increases following ADSC treatment. The dual-luciferase reporter gene assay confirmed that KLF5 directly binds to the CXCL12 promoter, enhancing its transcriptional activity. Knockdown of KLF5 in ADSCs reduced CXCL12 protein levels, and in vivo, it inhibited the ability of ADSCs to accelerate wound healing. Immunofluorescence staining further demonstrated reduced neovascularization in wounds treated with KLF5 knockdown ADSCs, highlighting KLF5 as a key player in the therapeutic effects of ADSCs.

EPCs play a central role in neovascularization through paracrine signaling and differentiation into mature endothelial cells [42]. Hyperglycemia in patients with diabetes leads to endothelial dysfunction [43, 44], reducing EPCs numbers and impairing their ability to mobilize, proliferate, adhere, and form new blood vessels [45, 46]. In our study, co-culture with ADSCs significantly enhanced EPC proliferation, migration, and tube formation in a hyperglycemic environment. However, these beneficial effects were diminished when KLF5 or CXCL12 were knocked down in ADSCs. Notably, adding recombinant CXCL12 rescued the impaired EPC function in co-cultures with KLF5-knockdown ADSCs, further confirming the critical role of the KLF5/CXCL12 pathway.

In conclusion, our findings highlight the importance of the KLF5/CXCL12 signaling axis in ADSC-mediated diabetic wound healing by enhancing the biological functions of EPCs. Targeting KLF5 represents a promising therapeutic strategy for diabetic wounds and may lead to the development of novel small-molecule therapies to improve wound healing outcomes.

## Conclusions

The local application of ADSCs significantly enhances diabetic wound healing, with KLF5 playing a pivotal role by promoting neovascularization through the upregulation of CXCL12. Increased CXCL12 expression stimulates EPC proliferation, migration, and vasculogenic capacity, all critical for effective wound repair. These findings offer valuable insights into the development of new therapeutic strategies targeting the KLF5/CXCL12 pathway for improved treatment of diabetic wounds.

## Supplementary Information

Supplementary material 1.

Supplementary material 2.

Supplementary material 3.

Supplementary material 4.

Supplementary material 5.

## Data Availability

All data and related analyses are included in this published article. All other data are available from the corresponding author upon request.

## Abbreviations

ADSCs: Adipose-derived stem cells
AGEs: Advanced glycation end products
BP: Biological processes
BSA: Bovine serum albumin
CC: Cellular components
ChlP: Chromatin immunoprecipitation
CPM: Counts per million
CXCL12: C-X-C motif chemokine 12
CXCR4: C-X-C motif chemokine receptor 4
CXCR7: C-X-C motif chemokine receptor 7
DAPI: 4′,6-Diamidino-2-phenylindole
DMEM: Dulbecco’s modified Eagle medium
DFUs: Diabetic foot ulcers
EBF2: Early b-cell factor 2
EBM: Endothelial cell basal medium-2
EGR4: Early growth response 4
EP: Eppendorf tube
EPCs: Endothelial progenitor cells
FBS: Fetal bovine serum
EDTA: Ethylenediaminetetraacetic acid
GAPDH: Glyceraldehyde-3-phosphate dehydrogenase
GO: Gene Ontology
h: Hour
IHC: Immunohistochemistry
HG: High glucose
LG: Low glucose
kb: Kilobase pairs
KEGG: Kyoto encyclopedia of genes and genomes
KLF5: Kruppel-like factor 5
kDa: Kilodalton
MAPK: Mitogen-activated protein kinase
MF: Molecular functions
min: Minute
MSCs: Mesenchymal stem cells
NRS: Neovascularization-related signature
OD: Optical density
OS: Oxidative stress
PAGE: Polyacylamide gel electrophoresis
PBS: Phosphate-buffered saline
PCR: Polymerase chain reaction
PI3K-Akt: Phosphatidylinositol 3-kinase-protein kinase B
PPI: Protein–protein interaction
PVDF: Polyvinylidene fluoride
rcf: Relative centrifugal force
RIN: RNA integrity number
RNA-seq: RNA sequencing
RT-qPCR: Quantitative reverse transcription PCR
ROS: Reactive oxygen species
rpm: Revolutions per minute
sec: Second
SDS: Sodium dodecyl sulphate
SPSS: Statistical Package for Social Science
shRNA: Short hairpin RNA
TBS: Tris-buffered saline
WB: Western blot
