# Supplementary figures and images for: KLF5 enhances CXCL12 transcription in adipose-derived stem cells to promote endothelial progenitor cells neovascularization and accelerate diabetic wound healing
Source: Cell Mol Biol Lett. 2025 Mar 4;30:24. doi: 10.1186/s11658-025-00702-0 (PMC11877965; doi:10.1186/s11658-025-00702-0)

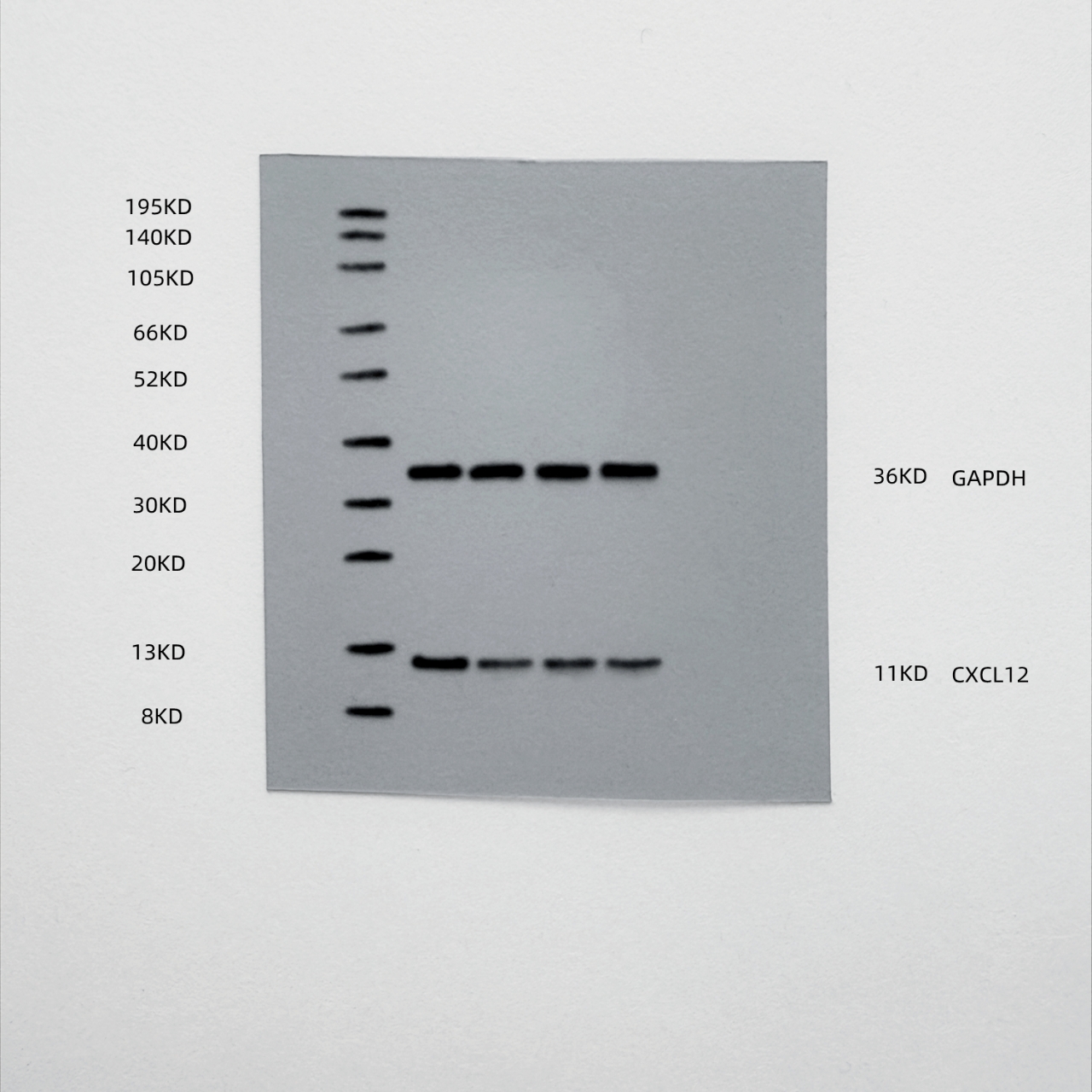

Supplement: Supplementary file 1 — Supplementary material 1. [file 11658_2025_702_MOESM1_ESM.jpg]

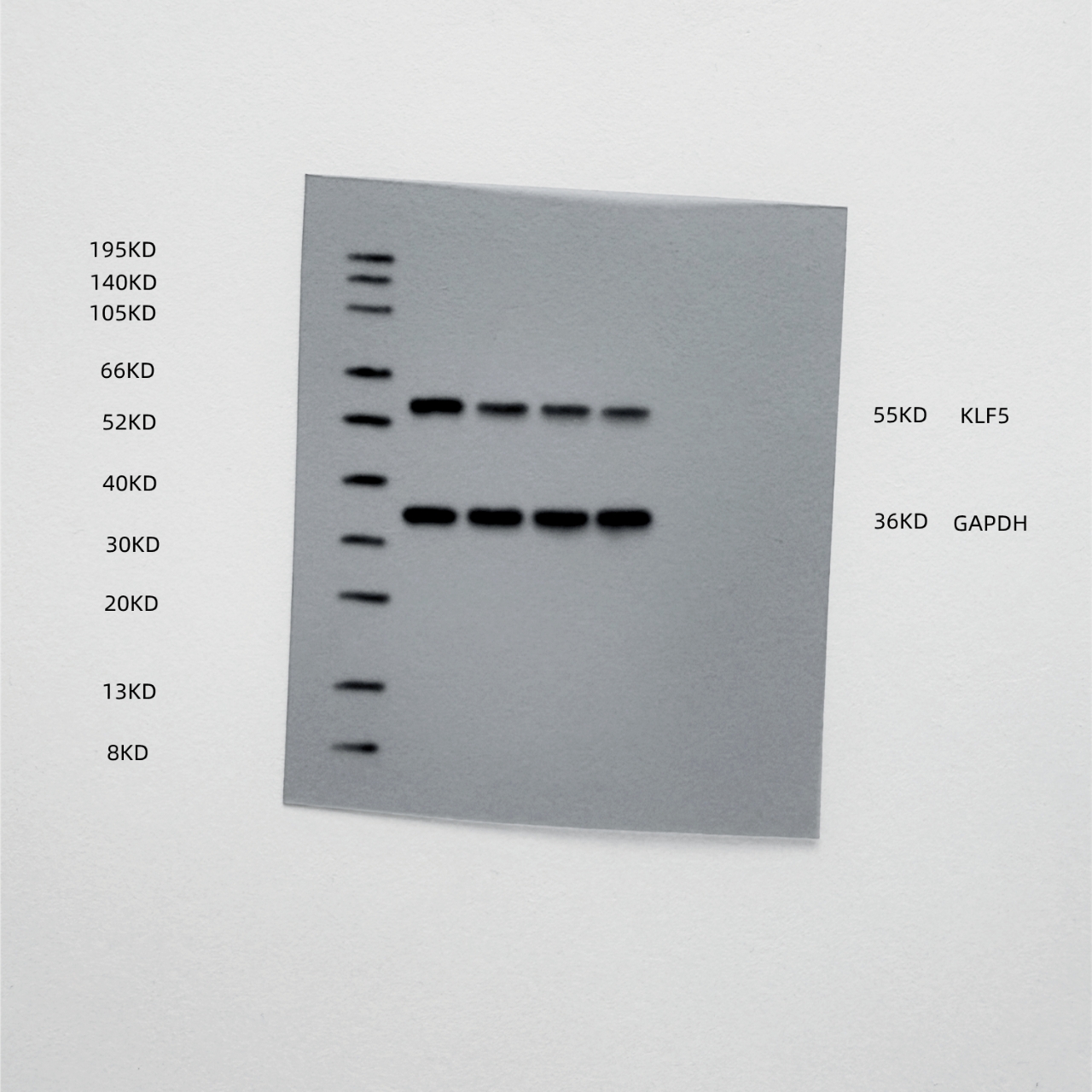

Supplement: Supplementary file 2 — Supplementary material 2. [file 11658_2025_702_MOESM2_ESM.jpg]

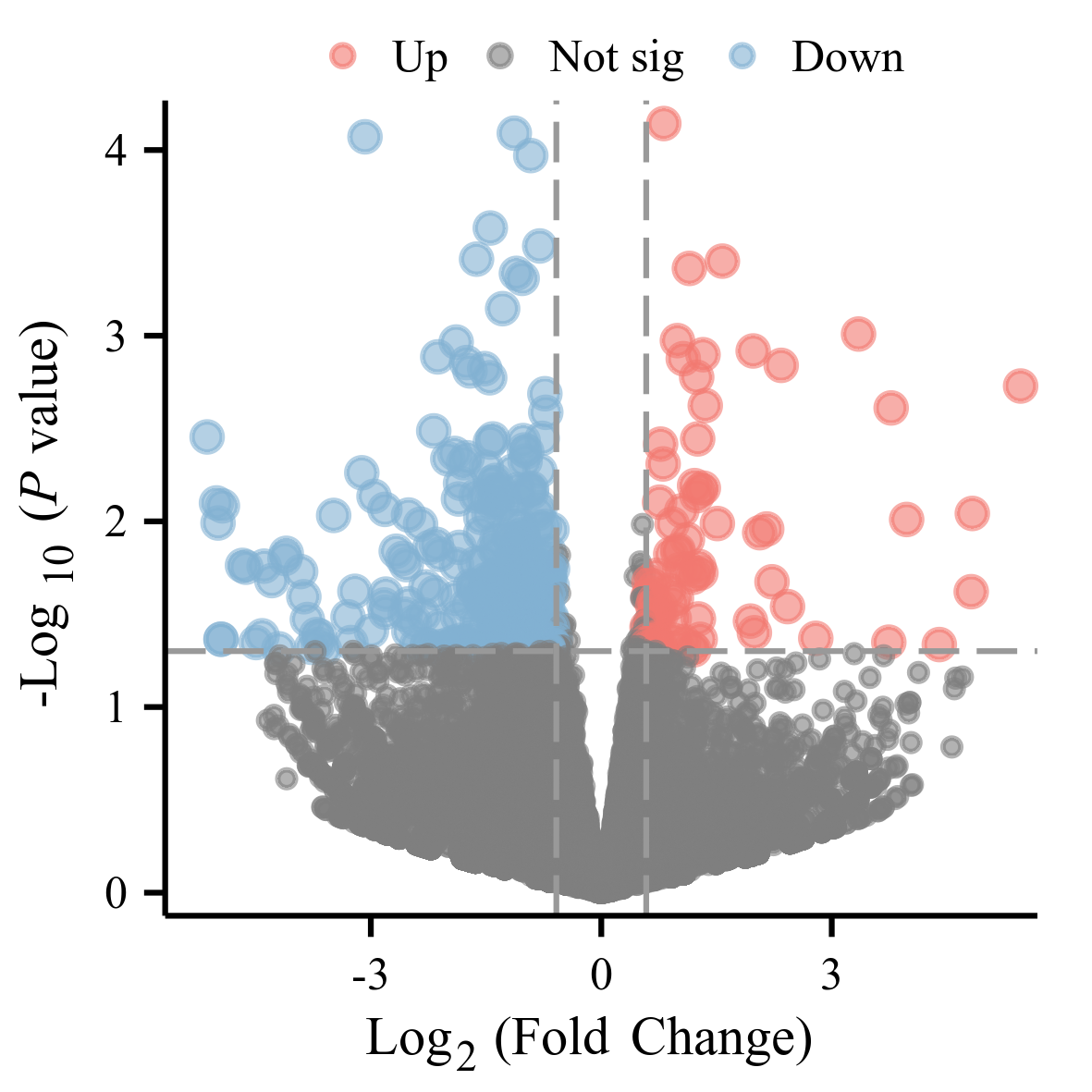

Supplement: Supplementary file 5 — Supplementary material 5. [file 11658_2025_702_MOESM5_ESM.zip › Supplementary material 5/1.1deg Volcano diagram fc-0.585.tiff]

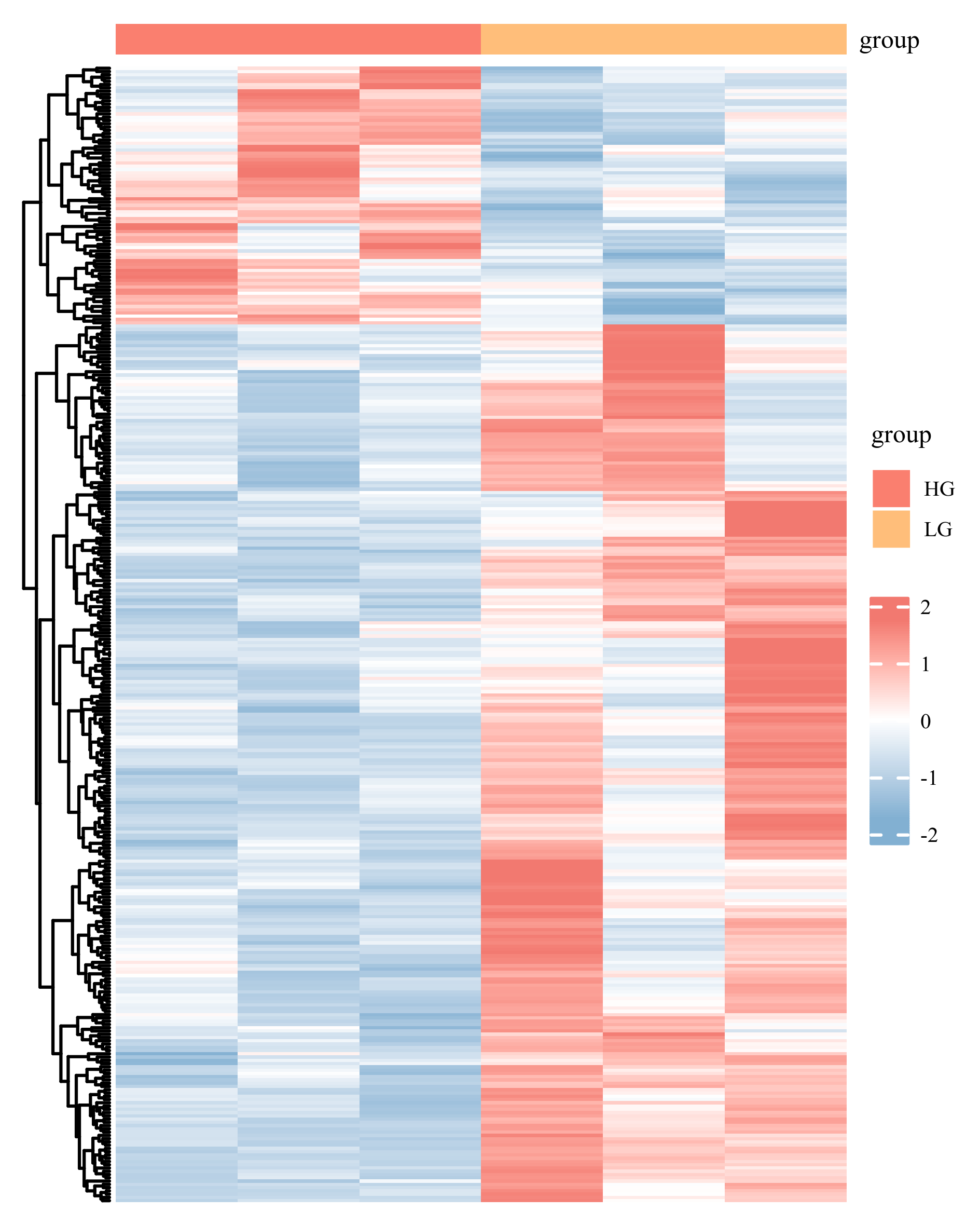

Supplement: Supplementary file 5 — Supplementary material 5. [file 11658_2025_702_MOESM5_ESM.zip › Supplementary material 5/2.hg_lg_deg Heat map.tiff]

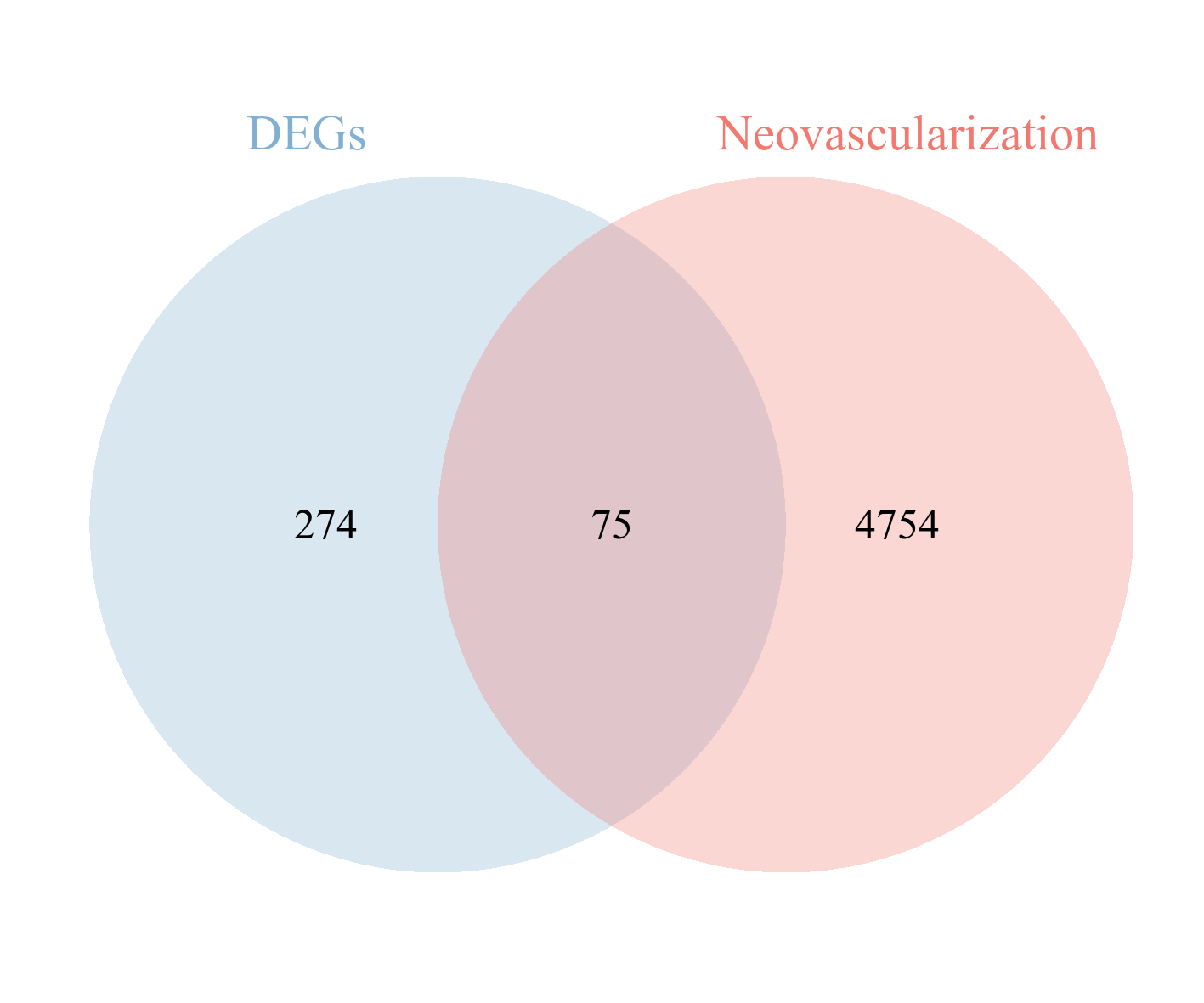

Supplement: Supplementary file 5 — Supplementary material 5. [file 11658_2025_702_MOESM5_ESM.zip › Supplementary material 5/3.1deg_neovascular Wayne chart.tiff]

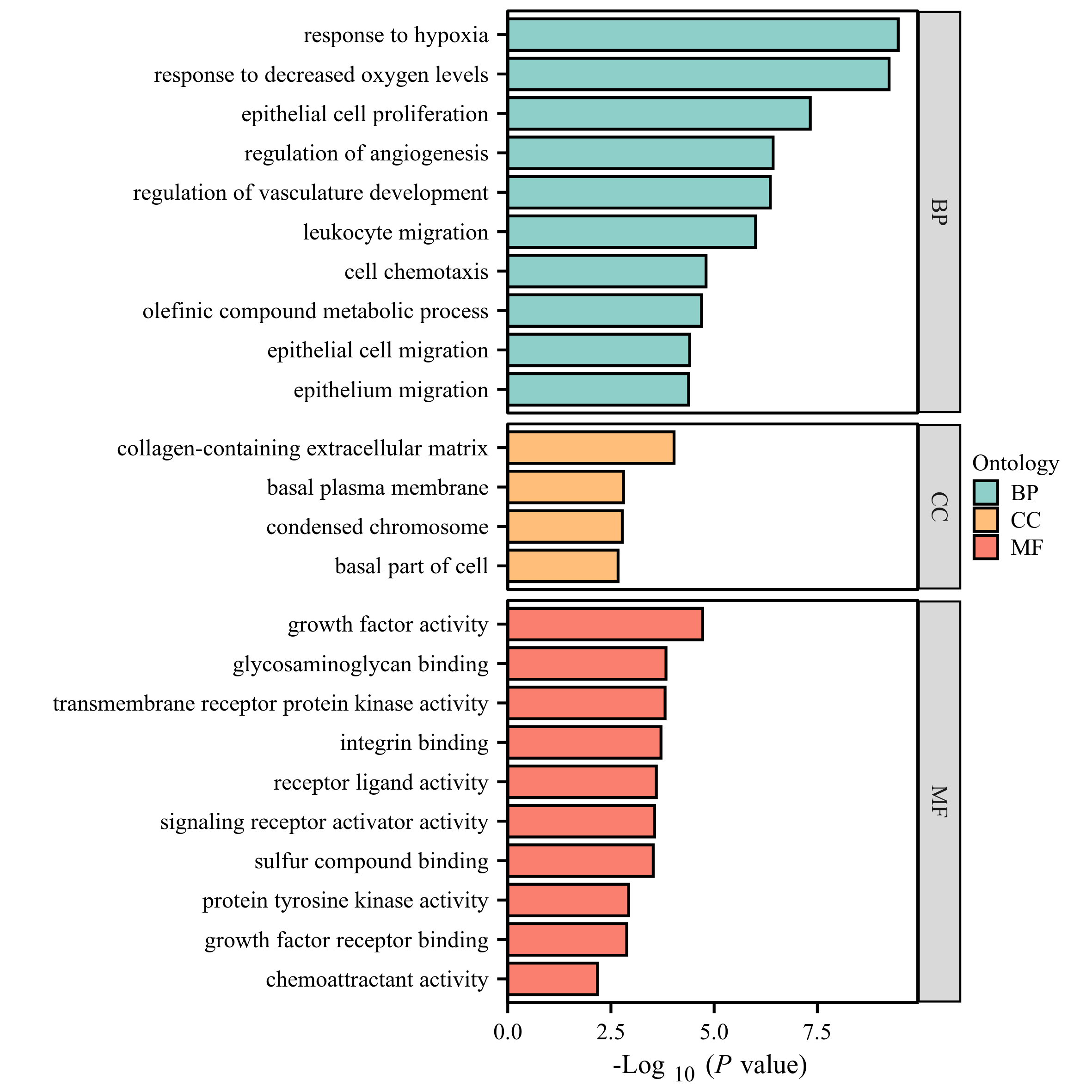

Supplement: Supplementary file 5 — Supplementary material 5. [file 11658_2025_702_MOESM5_ESM.zip › Supplementary material 5/4.2deg_neovascular_GO Bar chart.tiff]

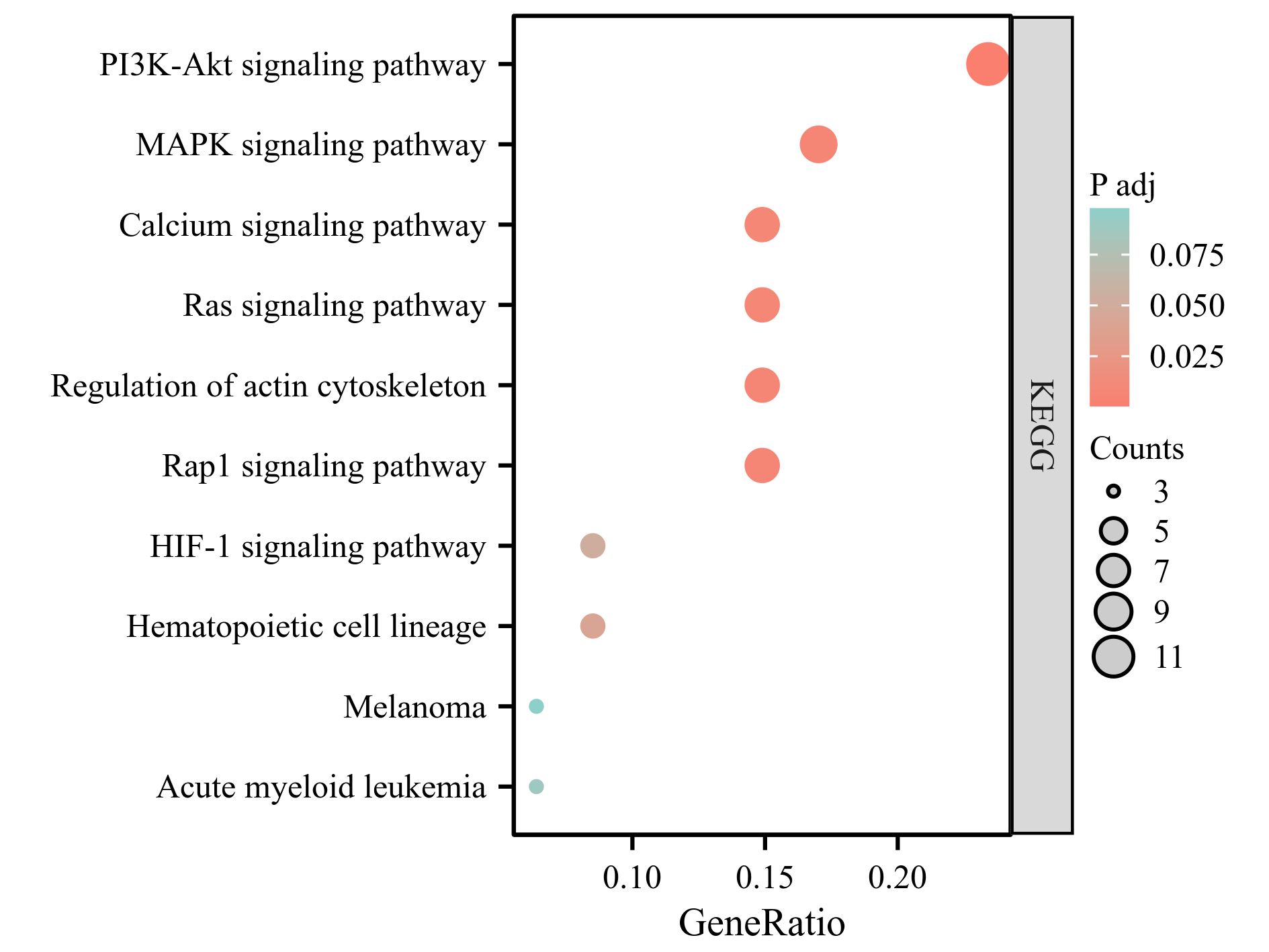

Supplement: Supplementary file 5 — Supplementary material 5. [file 11658_2025_702_MOESM5_ESM.zip › Supplementary material 5/4.3deg_neovascular_KEGG Bubble chart.tiff]

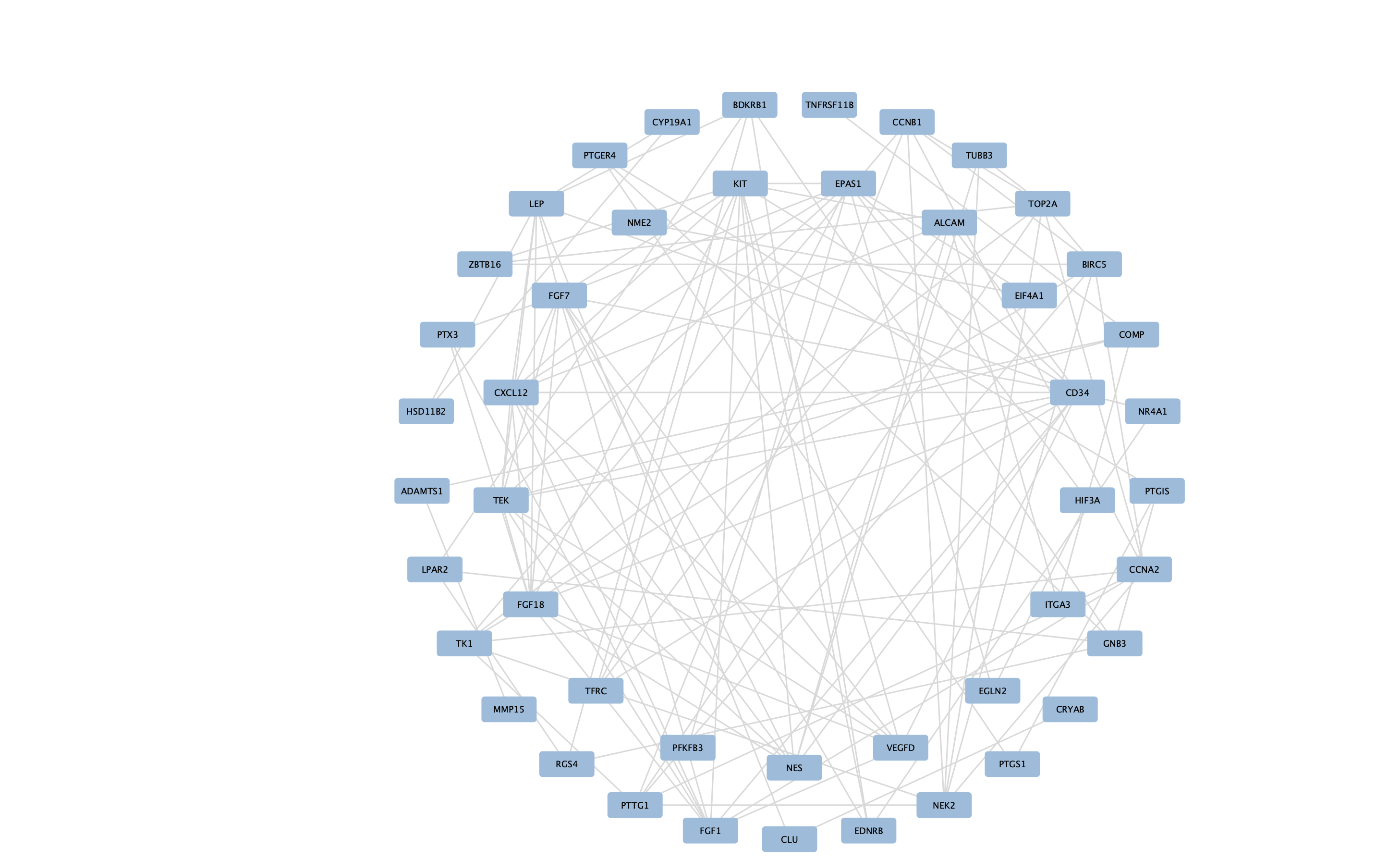

Supplement: Supplementary file 5 — Supplementary material 5. [file 11658_2025_702_MOESM5_ESM.zip › Supplementary material 5/5.0deg_neovascular_ppi.png]

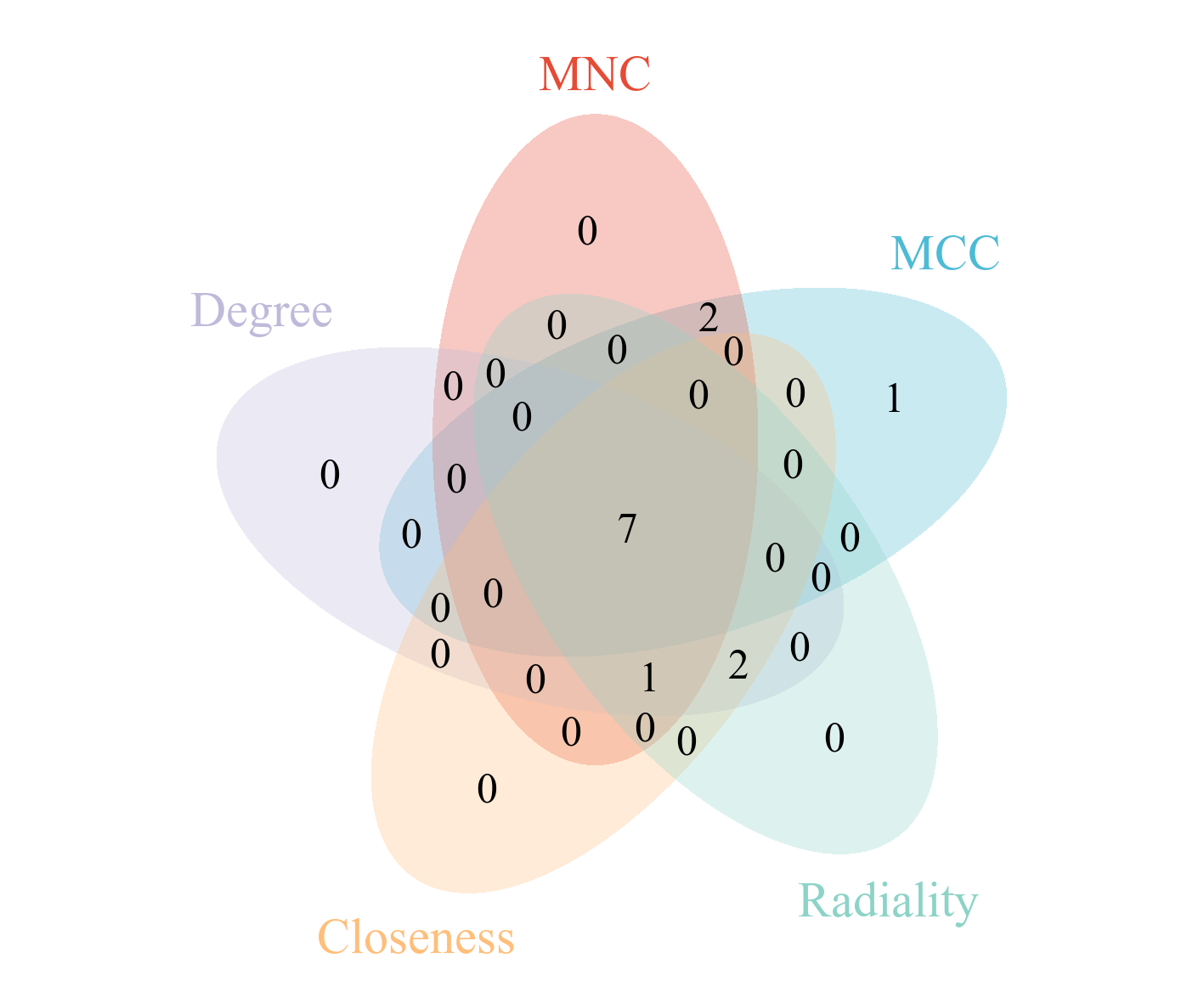

Supplement: Supplementary file 5 — Supplementary material 5. [file 11658_2025_702_MOESM5_ESM.zip › Supplementary material 5/5.8hubgene_ppi Wayne chart.tiff]
