# Supplementary material for: KLF5 enhances CXCL12 transcription in adipose-derived stem cells to promote endothelial progenitor cells neovascularization and accelerate diabetic wound healing
Source: Cell Mol Biol Lett. 2025 Mar 4;30:24. doi: 10.1186/s11658-025-00702-0 (PMC11877965; doi:10.1186/s11658-025-00702-0)
Supplement: Supplementary file 5 — Supplementary material 5. [file 11658_2025_702_MOESM5_ESM.zip › Supplementary material 5/renamed_03b65.docx]

xiuyingshan@fjmu.edu.c

No. 20, Chazhong Road

Fuzhou, Fujian, 350000

P. R. China

Jan 16, 2025

Dear Editors,

Thank you and the reviewers for the detailed and constructive feedback on our manuscript titled “KLF5 Enhances CXCL12 Transcription in Adipose-Derived Stem Cells to Promote Endothelial Progenitor Cells Neovascularization and Accelerate Diabetic Wound Healing” (Manuscript ID:CMBL-D-24-01380R2). We appreciate the time and effort the reviewers have taken, and we have made revisions accordingly. Below is a point-by-point response to the comments from each reviewer.

**Reviewer #1:**

1. Comment : Bigger font size in Graphical Abstract recommended.

Response: The font size in the Graphical Abstract has been increased for better readability.

2. Comment : Please define “DFU” at first mention (Page 4, line 9).

Response: We have defined “DFU” (Diabetic Foot Ulcer) at its first mention in the revised manuscript.

3. Comment: Please remove “lead to” or “cause” to improve grammar (Page 4, line 30).

Response: We have revised the sentence to improve grammar by delete “cause”.

4. Comment: Please state in which model system the observation was made (Page 5, line 17).

Response: We have clarified in the manuscript that the observation was made using the diabetic rats.

5. Comment: Add catalogue numbers for materials.

Response: Catalogue numbers for all materials have been added to the Methods section.

6. Comment: Define abbreviations BP, CC, MF (Page 11, line 49).

Response: We have defined the abbreviations BP (biological processes), CC (cellular components), and MF (molecular functions) at their first mention.

7. Comment: Characterization of ADSCs: I suggest adding CD31 for immunophenotyping.

Response: We understand the importance of CD31 in certain areas, especially related to endothelial cells. However, we have observed that many high-impact articles on ADSC characterization do not include CD31. In our study, we followed commonly adopted protocols for ADSC characterization, which focus on markers like CD29 and CD90. For your reference, we have provided a list of selected markers and relevant literature in Supplementary Material 3 (PMID:32848141, 38434722, 38765787, 38261813, 37712174, 33537078, 37165618, 36967397, 36153544).

8. Comment: “These results suggest that KLF5 directly binds to the promoter of CXCL12 and activates its transcription.” As ELISA was performed to measure CXCL12 levels but no RT-qPCR, the term “transcription” should be changed to “secretion” (Page 14, line 11).

Response: We have changed the term “transcription” to “secretion” to accurately reflect the measurement of CXCL12 levels via ELISA. Additionally, we have listed several high-impact articles that have validated transcription factor binding at the protein level in Supplementary Material 4 for further reference (PMID:37062845, 24925061, 33754039, 34893086, 38637831).

9. Comment: The figure panels (D, E, F, G in Fig. 1, 3B-D, 6B-D, and 8) are too small.

Response: We have resized Figures 1 (D, E, F, G), 3B-D, 6B-D, and 8 to improve readability.

**Reviewer #2:**

1. Comment: Please define “DFU” at first mention.

Response: “DFU” has been defined at its first mention in the manuscript.

2. Comment: Can you please adjust figures 3D and 6D? They look awfully similar, and the current coloring makes it hard to distinguish groups.

Response: We have adjusted the colors in Figures 3D and 6D to improve the distinction between groups.

3. Comment: String analysis in Fig. 1 is unnecessary for clarity. Please place it in supplemental and adjust the size of other panels to improve readership.

Response: The string analysis has been moved to the supplementary materials, and the size of the panels in Figure 1 has been adjusted for better clarity.

4. Comment: The discussion would benefit from an explanation of how microenvironmental glucose links to KLF5 regulation. Is there previous literature already?

Response: We have expanded the discussion to include an analysis of the potential mechanism linking microenvironmental glucose to KLF5 activity. Specifically, we have included a summary of studies indicating that transcription factors such as FOXO1 may regulate KLF5 expression by binding to its promoter under conditions of metabolic stress, such as high glucose levels (PMID: 33539225).

**Reviewer #3:**

1. Comment: Please provide a list of differentially expressed genes corresponding to Figures 1A, 1B, 1C, and 1D with their GO terms.

Response: We have included the list of differentially expressed genes and their GO terms in the Supplementary Materials 5, as suggested.

2. Comment: The RNA-seq raw fastq files should be deposited to GEO-NCBI, and the accession number should be provided.

Response: The RNA-seq raw fastq files have been deposited to GEO-NCBI, and the BioProject accession number is PRJNA1209402. The accession number is now included in the manuscript, and the data can be accessed at the following link: <https://dataview.ncbi.nlm.nih.gov/object/PRJNA1209402>.

We believe these revisions have improved the manuscript, and we sincerely appreciate the constructive comments and suggestions provided by the reviewers. We look forward to your feedback and hope for a positive response.

If you require any further information or additional changes, please do not hesitate to contact us.

Yours sincerely,

Xiuying Shan

On behalf of the authors
